# Cytokine response to the RSV antigen delivered by dendritic cell-directed vaccination in congenic chicken lines

**DOI:** 10.1186/s13567-017-0423-8

**Published:** 2017-04-05

**Authors:** Jitka Mucksová, Jiří Plachý, Ondřej Staněk, Jiří Hejnar, Jiří Kalina, Barbora Benešová, Pavel Trefil

**Affiliations:** 1 BIOPHARM, Research Institute of Biopharmacy and Veterinary Drugs, Jílové U Prahy, Czech Republic; 2 Institute of Molecular Genetics, Academy of Sciences of the Czech Republic, Prague, Czech Republic; 3 Institute of Microbiology, Academy of Sciences of the Czech Republic, Prague, Czech Republic

## Abstract

Systems of antigen delivery into antigen-presenting cells represent an important novel strategy in chicken vaccine development. In this study, we verified the ability of Rous sarcoma virus (RSV) antigens fused with streptavidin to be targeted by specific biotinylated monoclonal antibody (anti-CD205) into dendritic cells and induce virus-specific protective immunity. The method was tested in four congenic lines of chickens that are either resistant or susceptible to the progressive growth of RSV-induced tumors. Our analyses confirmed that the biot-anti-CD205-SA-FITC complex was internalized by chicken splenocytes. In the cytokine expression profile, several significant differences were evident between RSV-challenged progressor and regressor chicken lines. A significant up-regulation of IL-2, IL-12, IL-15, and IL-18 expression was detected in immunized chickens of both regressor and progressor groups. Of these cytokines, IL-2 and IL-12 were most up-regulated 14 days post-challenge (dpc), while IL-15 and IL-18 were most up-regulated at 28 dpc. On the contrary, IL-10 expression was significantly down-regulated in all immunized groups of progressor chickens at 14 dpc. We detected significant up-regulation of IL-17 in the group of immunized progressors. LITAF down-regulation with iNOS up-regulation was especially observed in the progressor group of immunized chickens that developed large tumors. Based on the increased expression of cytokines specific for activated dendritic cells, we conclude that our system is able to induce partial stimulation of specific cell types involved in cell-mediated immunity.

**Electronic supplementary material:**

The online version of this article (doi:10.1186/s13567-017-0423-8) contains supplementary material, which is available to authorized users.

## Introduction

Generation of de novo adaptive responses, including responses to vaccines, is primarily elicited by dendritic cells (DC), specialized leukocytes adapted for antigen capture, processing and presentation to T lymphocytes. Knowledge of these cells in a target species is therefore crucial in finding the most effective means of vaccination. The key role of T cell-mediated responses to cancer has been established in several models [1]. The antitumor immune response relies on DC, which act as professional antigen-presenting cells (APC). Altered DC function is common in tumors producing soluble factors—cytokines—with immunosuppressive activity [2, 3]. DC express, among other, the CD205 (Ly75, DEC205) molecule that functions as an endocytic receptor involved in the uptake of extracellular antigens. In the chicken, the presence of this molecule was also confirmed, along with its endocytic properties [4]. Importantly, DC could be targeted with antigen-conjugated monoclonal antibodies specific for CD205, which are then efficiently internalized, processed in the endosomal compartment, and presented to both major histocompatibility complex I (MHC I) and MHC II molecules [5].

In this study, we used a monoclonal antibody (anti-CD205) for direct antigen delivery. This strategy of activating different DC populations by direct in vivo targeting of their surface receptors has been pioneered by Steinman and Nussenzweig, who used antigen coupling to antibodies to target receptors on DC surfaces [6–8]. We used genetic fusion of the antigen with streptavidin (SA), which in its tetrameric form binds a biotinylated antibody targeting a surface receptor on the APC. Thus prepared complexes can deliver immunogens into DC through endocytosis with the selected surface receptor, enabling antigen processing and presentation, and leading to induction of adaptive immunity [9, 10].

We applied this novel vaccination approach to our previously described model system of inbred lines resistant (CB, CB.RI; regressors) or susceptible (CC, CC.RI; progressors) to progressive growth of Rous sarcoma virus (RSV)-induced tumors [2, 3]. RSV harbors the oncogene v-*src*, which is responsible for cell transformation [11]. Previous studies using the model system of Prague congenic lines established a decisive role of the B-F—chicken MHC class I—genes in the ability to regress RSV-induced tumors [12]. The immune-based mechanism of tumor regression in this experimental system has also been demonstrated. Both v-src and RSV envelope (env) proteins serve as antigens in particular chicken lines [13–16]. On the contrary to the MHC of typical mammals, there is only a single dominantly expressed class I molecule in the chicken MHC, which is of crucial importance for the function of particular haplotypes [17, 18]. Dominantly expressed class I (B-F) molecules of the B-F12 and B-F4 haplotypes of congenic lines CB and CC, respectively, have been analyzed in detail as to the binding capacity of predicted peptides derived either from the v-src oncogene or env-encoded protein products of RSV [19–21]. Furthermore, coevolution of the B-F and peptide transporter genes (TAP) within the above-mentioned haplotypes has been established [20]. Thus, the ability of certain class I (B-F) molecules to specifically bind the RSV-derived peptides correlates precisely with the outcome of the RSV-induced tumors—regression/progression [19, 22]. The molecular mechanism of B-F (class I) and TAP interaction is well understood [20]. On the contrary, the function of the B-G (class IV) molecules and their possible interaction with the B-F (class I) molecules is still not clear [23], despite a wealth of experimental evidence suggesting the role of the B-G molecules in modulation of some immune based phenomena [24–28].

In this study, we describe a new system of antigen delivery into chicken DC and assess its potential for vaccination of chickens. In order to get some insight into the cellular and molecular mechanisms, we paid particular attention to changes in the cytokine profile after vaccination and immune challenge.

## Materials and methods

For preparation of anti-chCD205 and for construction, expression and purification of the recombinant SA-RSV fusion proteins, see Additional files 1, 2, 3.

### Experimental animals

All immunization and tumor induction experiments were carried out with the highly inbred chicken lines CB (B12/B12), CB.RI (B12r1/B12r1) and CC (B4/B4), CC.RI (B4r1/B4r1) maintained at the Institute of Molecular Genetics, Prague. Recombinant haplotype B12r1 is composed of B-F12 and B-G4 genes whereas the haplotype B4r1 is reciprocal being composed of B-F4 and B-G12 genes [29]. All chicken lines were free of exogenous avian leukosis viruses. All procedures were conducted in accordance with the EU Directive 2010/63/EU for animal experiments, comply with the ARRIVE guidelines and with the Guide for the Care and Use of Laboratory Animals and were approved by the Animal Care and Use Committee of the Academy of Sciences of the Czech Republic.

### Specificity of the monoclonal antibody anti-chCD205 and its internalization

For Western blotting, the recombinant proteins CD205B and CD205F were separated on Tris-Tricine SDS PAGE and transferred to the nitrocellulose membrane (Immobilon-P, GE Healthcare). Membranes were blocked with 5% nonfat milk in PBS containing 0.05% Tween-20 (PBST) for 1 h at room temperature, incubated with primary antibodies in PBST containing 5% nonfat milk for 1 h at room temperature prior to incubation with horseradish peroxidase-conjugated goat anti-mouse antibody (GE Healthcare) diluted in 5% nonfat milk PBST for 1 h at room temperature. Detection was carried out using a SuperSignal West Femto Maximum Sensitivity Substrate chemiluminescence reagent kit (Pierce, Thermo Fisher Scientific).

For the flow cytometry, peripheral blood mononuclear cells (PBMC) were isolated from chicken heparinized blood samples. Pieces of freshly isolated chicken spleen were crushed on a steel sieve. Splenocytes as well as PBMC were washed and centrifuged in a 1.077 density Histopaque gradient (Sigma-Aldrich) to remove nucleated erythrocytes. The monoclonal antibodies against chicken antigens CD4 (MCA2164F, AbDSerotec, USA), CD8a (MCA2166PE, AbDSerotec, USA), Bu1 (8395-08, AV20, Southern Biotech, USA), MHCII (8350-01, 2G11, Southern Biotech, USA), KUL01 (8420-02, Southern Biotech, USA), TCRλδ (CON5-CZ, TCR1, Exbio, Czech Republic), and putative CD11c (clone 8F2, a generous gift from Prof. Kaspers, University of Munich) were used. Conjugated complexes CD205-biot (clone 104-2C4/D8) and SA-FITC (SA1001, Invitrogen, USA) as well as secondary antibodies rabbit anti-mouse IgG-APC (SAB1426, Gentaur), mouse anti-chicken IgG-FITC (clone G-1, Southern Biotech, USA), and goat anti-mouse IgM-PE (sc-3768, SantaCruz Biotech) were employed.

For internalization, chicken spleen cells were incubated at 1 × 10^6^ cells per well at 4 °C with 1.5 µg/mL of antibody against CD205-biot. The cells were then washed and incubated with SA-FITC conjugate for 1 h at 4 °C. After that, the cells were incubated either at 40 or 4 °C for 1 or 2 h. The reduction of mean fluorescence intensity (MFI) of the cell surface-bound anti-chCD205 monoclonal antibody after endocytosis was calculated as 100 − ((MFI biot-mAb + FITC-SA 40 °C) − (MFI biot-control Ig + FITC-SA 4 °C)/(MFI biot-mAb + FITC-SA 4 °C) − (MFI biot-control Ig + FITC-SA 4 °C)) × 100. The cells were analyzed using a FacsCalibur device (Becton–Dickinson) and FlowJo software (TreeStar).

### Immunization of chickens

Chickens were immunized with a dose of 100 nmol of the mixture of SA-RSV tetramers complexed with 200 nmol anti-CD205 in the presence of 200 µg poly I:C (Sigma Aldrich, P1530) per chicken. The mixture of SA-RSV was composed of equal amounts of tetramers SA-vsrcA; SA-vsrcB; SA-ENVoep; SA-ENVly; SA-POL; SA-GAG—approximately 17 nmol (~4 µg) of each per chicken. The immunization dose was adjusted as described previously [9, 10]. The control group was mock-immunized with 200 µg of poly I:C per chicken.

Chickens were immunized with 0.2 mL of appropriately diluted freshly prepared antigenic solution subcutaneously (sc) into the left wing web (needle 23G × 25 mm). Immunization was repeated three times, first at the age of three weeks and then with two consecutive boosts at 7-day intervals.

### RSV challenge, tumor monitoring

The Prague strain of RSV (PR-RSV-C) was used [30] as tumor inducing challenge. Chickens were sc inoculated with a dose of to 100 (CB, CB.RI) or 20 (CC, CC.RI) focus-forming units (FFU) in 0.2 mL of cultivation medium (DMEM) into the outer area of pectoral muscle one week after the last immunization. At the end of the defined experimental period (28–35 days post-challenge, dpc), chickens were sacrificed, tumors were excised and weighed with 0.1 g precision. Tumors were then arbitrarily divided into two groups: small tumors, i.e. tumors up to 5 g (including completely regressed tumors) in the regressor lines CB and CB.RI, and tumors up to 20 g in the progressor lines CC and CC.RI; large tumors, i.e. tumors over 5 g in the CB and CB.RI, and over 20 g in the CC and CC.RI lines.

### RNA isolation, reverse transcription

Leukocytes from whole blood were obtained using Histopaque-1077 (Sigma) centrifugation. Total RNA from chicken leukocytes was isolated using Tri Reagent according to the manufacturer’s protocol (MRC, USA; Thermo Scientific, USA). RNA integrity number (RIN) was measured using capillary electrophoresis performed in Agilent Bioanalyzer 2100 with RNA 6000 Nano Assay (Agilent Technologies, CA, USA) and was adjusted to be higher than 6.5 in all samples selected for the analyses. RNA samples (400 ng/µL) were reverse transcribed to cDNA using a Transcriptor High Fidelity cDNA Synthesis Kit (Roche) according to the manufacturer’s instructions.

### Gene expression analysis

Gene expression analysis was performed in BioMark (Fluidigm, CA, USA), which enables a large number of real-time PCR (RT-PCR) reactions in a single run. Before BioMark analysis, the samples were pre-amplified. The RT-PCR reactions were carried out in GE Dynamic Chip 96.96 in a BioMark HD System (Fluidigm). 5 µL of sample premix consisted of 1 µL of 20 × diluted pre-amplified cDNA, 0.25 µL of 20 × DNA Binding Dye Sample Loading Reagent (Fluidigm), 2.5 µL of FastEvaGreen Supermix (Bio-Rad), 0.1 µL of 4 × diluted ROX (Invitrogen, USA) and 1.15 µL of RNase/DNase-free water. Each 5 µL assay premix consisted of 2.5 µL of 10 µM primers and 2.5 µL of DA Assay Loading Reagent (Fluidigm). The conditions of RT-PCR were the following: 95 °C for 3 min, 30 cycles of 95 °C for 5 s, and 60 °C for 20 s. The melting curve analysis was then performed. Primers were designed using Primer3 software or described sequences were used [31, 32] (Additional file 4). The GAPDH and 28S reference genes were selected out of the reference gene candidates by Normfinder (GenEx, Sweden). Each sample was analyzed in three technical replicates. The data were collected using BioMark 3.1.2. Data Collection software and analyzed by Fluidigm Real-Time PCR Analysis Software 4.1.2. (Fluidigm).

The cut-off value for Cq was set at 23 and values higher than that were replaced by the Cq value of 25. A few missing values were replaced by the highest value plus 2, then the data were recalculated into relative quantities and logarithms were applied. Data were normalized with GAPDH. The fold change in expression was calculated using the 2-Cq method for each sample and then expressed as the mean of all fold changes. The control was set as 100% and experimental samples were compared to the control.

### In vitro antigen restimulation and Multiplex cytokine assays

Chickens were sc immunized three times at seven day intervals with a dose of 100 nmol of the SA-RSV tetramers (equal amounts of tetramers SA-vsrcA; SA-vsrcB; 50 nmol of each) complexed with 200 nmol biotin-anti-CD205 in the presence of 200 µg poly I:C per chicken. At day 10 after the last immunization, PBMC were isolated and in vitro restimulated (250 000 cells/well) with SA-vsrcA + SA-vsrcB antigen (10 μg/ mL) and free SA (10 μg/ mL) as a control. Cells were cultivated at 37 °C in RPMI-1640 (Sigma) supplemented with 10% FBS (Gibco) and 1% Glutamine–Penicillin–Streptomycin (Sigma). 4 days after in vitro restimulation, the v-src-specific interleukin (IL-12, IL-2, IFNγ, IL-10) responses were measured using the Bio-Plex system in the culture supernatants. For each cytokine, the recombinant protein, capture antibody and detection antibody were obtained as complete kit (Kingfisher Biotech, USA; cat.nos. DIY0686C-003 IL-12; DIY0685C-003 IL-2; DIY0684C-003 IFNγ; DIY1118C-003 IL-10).

Culture supernatants from Ag-restimulation assays were collected at the indicated time. Cytokine profiles were then tested using Bio-Plex Pro Assays (BIO-RAD) according to the manufacturer’s instructions. Each cytokine was tested in a single bead assay to determine the optimal concentration of antibody detection. The microspheres were multiplexed and optimized for incubation times and Streptavidin-PE reporter signal. All assays were performed with the same matrix as the culture supernatants, calibration curves from recombinant cytokine standards were prepared with threefold dilution steps. Measurements and data analysis of all assays were performed with the Bio-Plex system in combination with the Bio-Plex Manager Software.

### Statistical analyses

The weights of individual tumors were compared using ANOVA with Fisher LSD test, Mann–Whitney test and *t* test. Data of gene expression were prepared in Genex 5.3.7 software (GenEx). The following analysis was done in SAS 9.4 software. Groups were compared by repeated three-way ANOVA. Contrasts were used for detailed comparison. Linear discriminant function analysis based on all analyzed genes was used to show separation of different groups. The cytokine expression profiles of RSV-challenged chicken groups were classified using methods of principal component analysis (PCA) [33] and linear discrimination analysis in XLSTAT software (StatSoft, Czech Republic).

## Results

### SA-RSV fusion proteins

**Figure 1:**
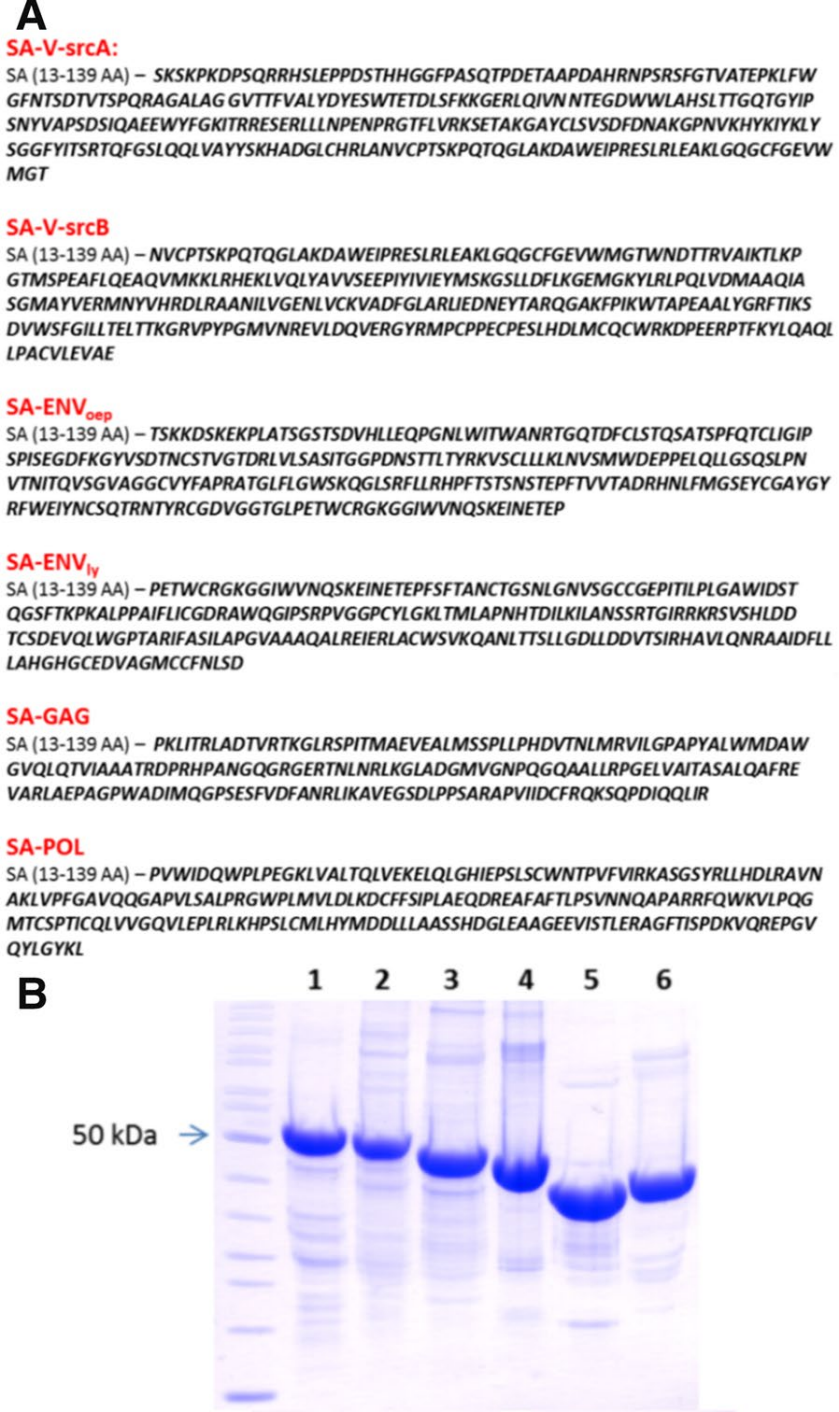
**Expression and purification of Ag-SA fusion proteins. A** Amino acid sequence of SA-RSV fusion protein. **B** Purified SA-RSV fusion protein (15 µg) eluted from the 2-Iminobiotin Agarose column was separated in Tris-Tricine SDS-PAGE gel and the proteins were visualized by Coomassie blue staining. 1—SA-V-srcA; 2—SA-V-srcB; 3—SA-ENVoep; 4—SA-ENVly; 5—SA-GAG; 6—SA-POL. All samples were heated to 100 °C for tetramer dissociation. Only the denatured monomeric forms of antigen streptavidin fusion proteins are visualized on the gel.

The complete sequence of the RSV antigens v-src, env, pol and gag were fused to the N- and C-terminus of the tetramerization core of streptavidin. The SA fusion with whole antigens v-src and env did not form stable tetramers due to their size. These two antigens were therefore split into two overlapping parts. In env, the signal, transmembrane and intracellular domain were also excluded (Figure 1A). All fusion proteins were produced in *E. coli* and purified close to homogeneity (Figure 1B). The fusion proteins with antigens fused to the C- terminal part of SA (SA-RSV) were isolated in higher purity and displayed longer stability than the N-terminus fusions (RSV-SA). The SA-RSV tetramers were therefore used for the vaccination study.

### Specificity and internalization capacity of the monoclonal antibody anti-chCD205

**Figure 2:**
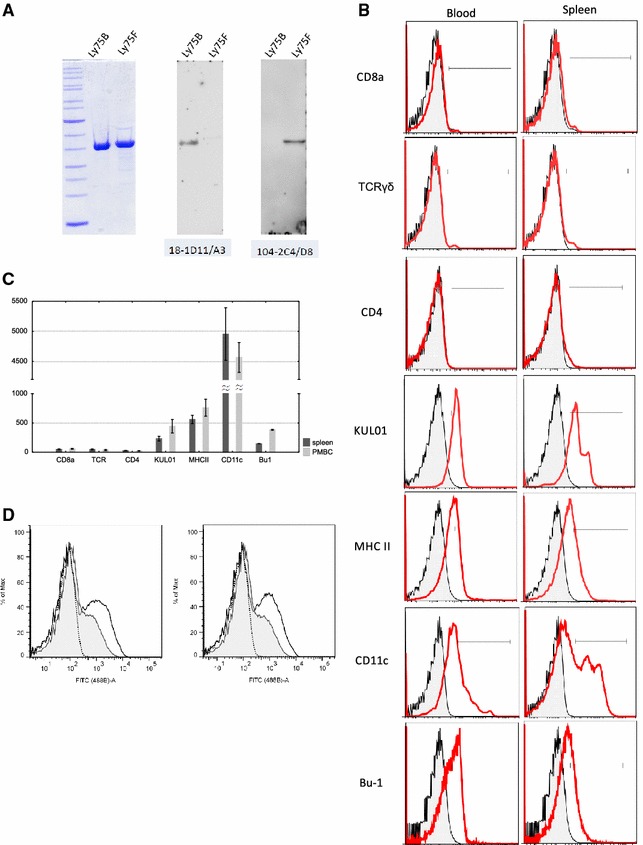
**Specific binding of anti-chCD205 and internalization of antigen delivery complex. A** The recombinant parts of chicken CD205 receptor used for monoclonal antibody preparation and the monoclonal antibody characterization. The CD205B and CD205F proteins were purified from inclusion bodies on DEAE Sepharose and separated on Tris-Tricine polyacrylamide gel stained by Coomassie blue (left). Monoclonal mouse anti-CD205 antibodies were analyzed using Western blots. Results of two representative detections of monoclonal anti-CD205 specific for CD205B or CD205F are shown. **B** CD205 expression on leukocytes. Splenocytes and peripheral leukocytes were stained with anti-chicken CD205 antibody and antibodies recognizing each of the indicated antigens. Histograms represent the CD205 fluorescence for cells gated for positive expression of the markers indicated at the left. The results of one representative of three experiments are shown. **C** Median fluorescence intensity (MFI) of CD205 stained cells and standard deviation. **D** Internalization of streptavidin-CD205-FITC complex by chicken primary splenocytes one (left) and two (right) hours of incubation in different temperatures. Dotted line plot non-labelled cells (biot—control IgG); Black line plot—FITC labelled cells incubated in 4 °C; Gray line plot—FITC labelled cells incubated in 40 °C.

The specificity of newly prepared anti-chCD205 monoclonal antibodies was tested by ELISA against recombinant antigens CD205B and CD205F (data not shown). The specificity of the positive clones was verified on Western blot, where the recombinant proteins CD205B and CD205F were detected using different clones of monoclonal antibodies (Figure 2A). For the purpose of antigen delivery, it was necessary to select antibodies recognizing natural surface receptor CD205 by flow cytometry on chicken splenocytes and blood cells. Flow cytometry data were gated to exclude cell debris according to the cell granularity and size (SSC/FSC parameters). CD205 was expressed at very low levels on T cell subsets of leukocytes (CD4^+^, TCRγδ^+^, and CD8a^+^) as well as splenocytes. As shown in Figures 2B and C, higher expression levels of CD205 were seen on KUL01^+^ CD11c^+^, MHCII^+^ cells and Bu1^+^ cells.

FITC-labeled cells incubated at 40 °C showed a substantial decrease of surface fluorescence intensity after 1 and 2 h of incubation, while the cells left incubated at 4 °C showed similar fluorescence to the initial analysis. MFI was reduced reaching 36.4% after 1 h and 26.8% after 2 h of incubation at 40 °C (Figure 2D). This indicates that the biot-anti-CD205-SA-FITC complex was internalized into cells and entered the low pH milieu of the cell endocytic apparatus as FITC dye is pH-sensitive [34].

### In vitro antigen restimulation

**Figure 3:**
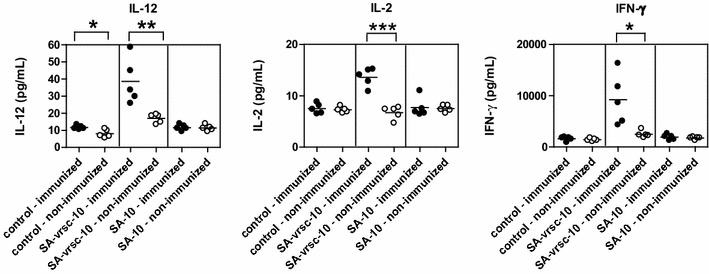
**In vitro Ag restimulation.** The v-src-specific interleukin (IL-12, IL-2, IFNγ, IL-10) responses were measured in chickens of the CB and CB.RI lines using the Bio-Plex system in the culture supernatants. Horizontal bars indicate the mean values. The results represent at least two independent experiments. Control (*n* = 5 + 5): non-restimulated control; SA-vsrc-10 (*n* = 5 + 5): samples restimulated with the mixture of tetramers SA-vsrcA and SA-vsrcB of immunized and non-immunized animals; SA-10 (*n* = 5 + 5): free SA restimulation control. Statistical significance determined by the Student’s *t* test: *p < 0.05; **p < 0.01; ***p < 0.001.

In order to verify the changed expression of the three major cytokines (IL-2, IL-12 and IFNγ), the in vitro restimulation of PBMC was performed. The levels of three cytokines detected in PBMC culture supernatants (Il-2, IL-12 and IFNγ) were significantly increased in the immunized groups that were restimulated with v-src antigen. On the contrary, the amount of IL-10 was at the same level as in PBMC restimulated with v-src or control antigen or in the group without restimulation respectively (Figure 3).

### RSV-induced tumor growth in the high (CB, CB.RI) and low (CC, CC.RI) responder chicken lines

**Figure 4:**
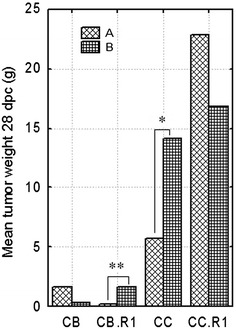
**Comparison of tumor size between control and immunized groups of chickens.** For statistical analysis, the non-immunized (**A**) and immunized (**B**) chickens of all inbred lines (CB, CB.RI, CC and CC.RI) were considered. Chickens of the regressor lines CB, CB.R1 and the progressor lines CC, CC.R1 were challenged with 100 FFU and 20 FFU of the RSV-PR-C respectively (for details see “Materials and methods”). Each column represents an average tumor size in the group of eight chickens (total number 64 chickens). *p < 0.05 non-parametric test (Mann–Whitney); **p < 0.01 both parametric (*t* test) and non-parametric test (Mann–Whitney).

As expected, the general pattern of the response to RSV challenge represented regression of relatively small tumors (≤2 g) in the CB and CB.RI lines, while progressive growth of tumors was observed in the CC and CC.RI lines even when challenged with a fivefold lower dose of RSV (Figure 4). The difference in the mean tumor weight 28 dpc between the regressor lines CB and CB.RI and progressor lines CC and CC.RI was highly significant (data not shown). However, on the contrary to expectations, we observed an exacerbating effect of immunization in the CB.RI (*p* < 0.01) and CC (*p* < 0.05) chickens (Figure 4). However, a potential protective effect of immunization can be seen in the CB and CC.RI lines, but these differences were not significant. Interestingly, the adverse effect of vaccination correlates with the B-G4 allel presented in both CB.R1 and CC chickens. However, a protective effect was seen in the CB (regressors) chicks with MHC-B haplotype composed of its canonical partners the B-F12 and B-G12, and also in the CC.R1 (progressors) chicks with the B-G12 allele present in the recombinant haplotype together with the B-F4—responsible for generally progressor phenotype (for composition of the MHC-B haplotypes see “Materials and methods”, and “Discussion”).

### Comparison of the cytokine profiles in RSV-challenged chickens of different congenic lines

**Figure 5:**
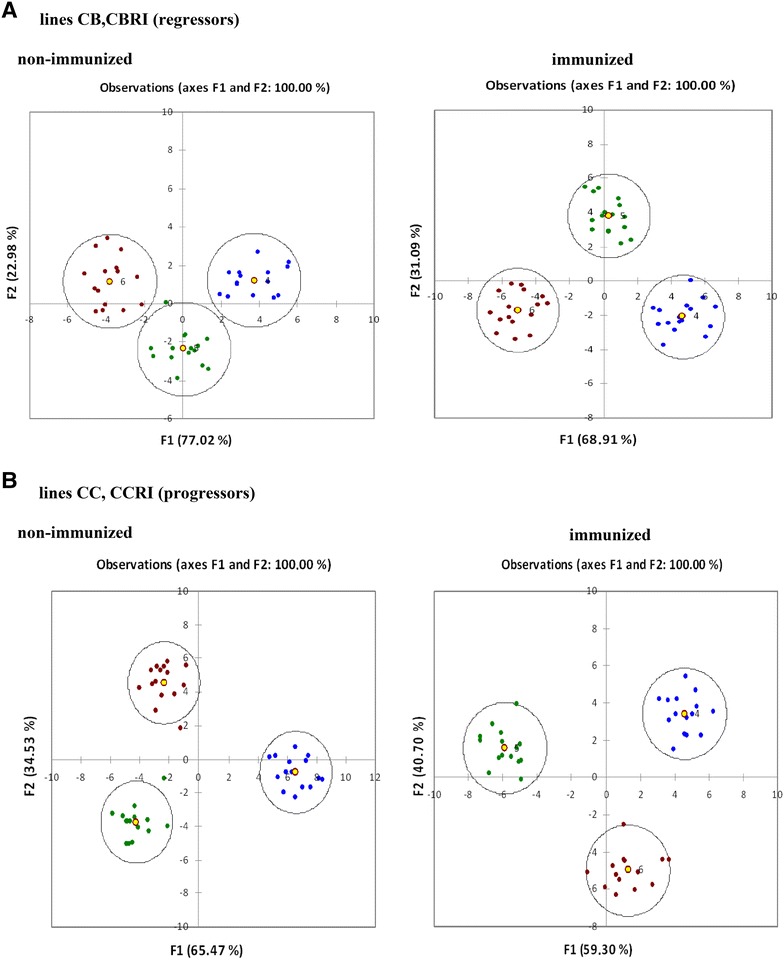
**Comparison of the kinetics of cytokine pattern in RSV challenged chickens of different congenic lines.** Classification of individual responses by PCA and discriminant analysis in immunized and non-immunized groups of regressors (**A**) and progressors (**B**). Samples were classified into three groups by using PCA (XLSTAT software). The first group (blue spots) includes samples taken on the time of challenge, the second group (green spots) includes samples obtained 14 days after challenge and the third group (brown spots) comprises samples obtained 28 days after challenge. F1 and F2 represent the two main components.

Expression of twenty genes (see Additional file 4) active in chicken blood cells that can influence the process of immune response and consequently the tumor growth were tested. To compare the kinetics of the cytokine profiles in RSV-challenged chickens, we pooled CB and CB.RI lines and CC and CC.RI lines into the groups of regressors and progressors, respectively. The multidimensional information on cytokine gene expression profiles was transformed into a 2-dimensional plot using PCA. Classification of the tested samples based on PCA results was confirmed by discriminant analysis, in which a score of 100% was reached for the confusion matrix. Based on the multidimensional gene expression profile, the samples of all experimental chicken groups were classified into three clusters (Figure 5) at each sampling time. In the group of regressors, the most prominent difference in multidimensional cytokine expression between the immunized and non-immunized groups was detected 14 dpc, while in the group of progressors the most prominent difference was seen 28 dpc. In the non-immunized groups (all times) of regressors there were significant similarities of clusters in comparison to immunized regressor groups. Interestingly, the multidimensional expression profiles in groups at time of challenge and 28 dpc (both non-immunized and immunized) were similar by F1 main component.

**Figure 6:**
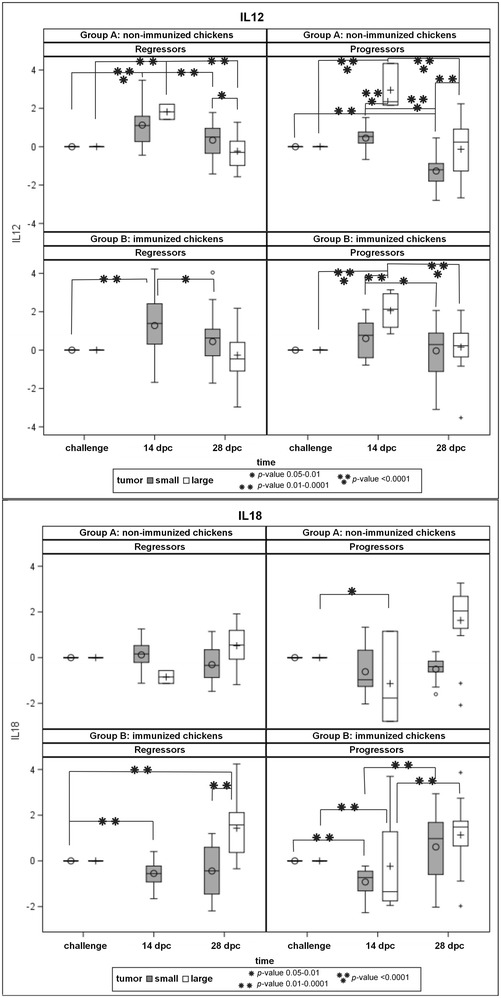
**Single cytokine profiles of selected genes in progressor and regressor groups of immunized and non-immunized chickens.** The expression value after challenge was normalized to the basic value expression in the day of challenge. For more cytokines, see Additional file 5.

When comparing single cytokine expression profiles between progressor and regressor immunized and non-immunized groups, several significant differences were found (see Additional file 5). A significant up-regulation of IL-2, IL-12, IL-15 and IL-18 expression was detected in immunized chickens of both regressor and progressor groups. Of these cytokines, IL-2 and IL-12 expression was most up-regulated 14 dpc, while IL-15 and IL-18 were most up-regulated 28 dpc (Figure 6; IL-12, IL-18). On the contrary, IL-10 expression was significantly down-regulated 14 dpc in all immunized groups of progressor chickens. Interestingly, we detected significant up-regulation of IL-17 in the group of immunized progressors and significant up-regulation of MIF and XCL1 in both groups of progressors 14 dpc. Surprisingly, down-regulation of LITAF, a putative chicken analog of TNFα, and up-regulation of iNOS were observed especially in the progressor group of immunized chickens that developed large tumors.

### The cytokine pattern correlates with the RSV-induced tumor status (regression/progression)

The PCA method and discrimination analysis were used for classification and discrimination of multimarker gene cytokine expression profiles of experimental chickens. Individual samples were distributed based on the tumor weight.

**Figure 7:**
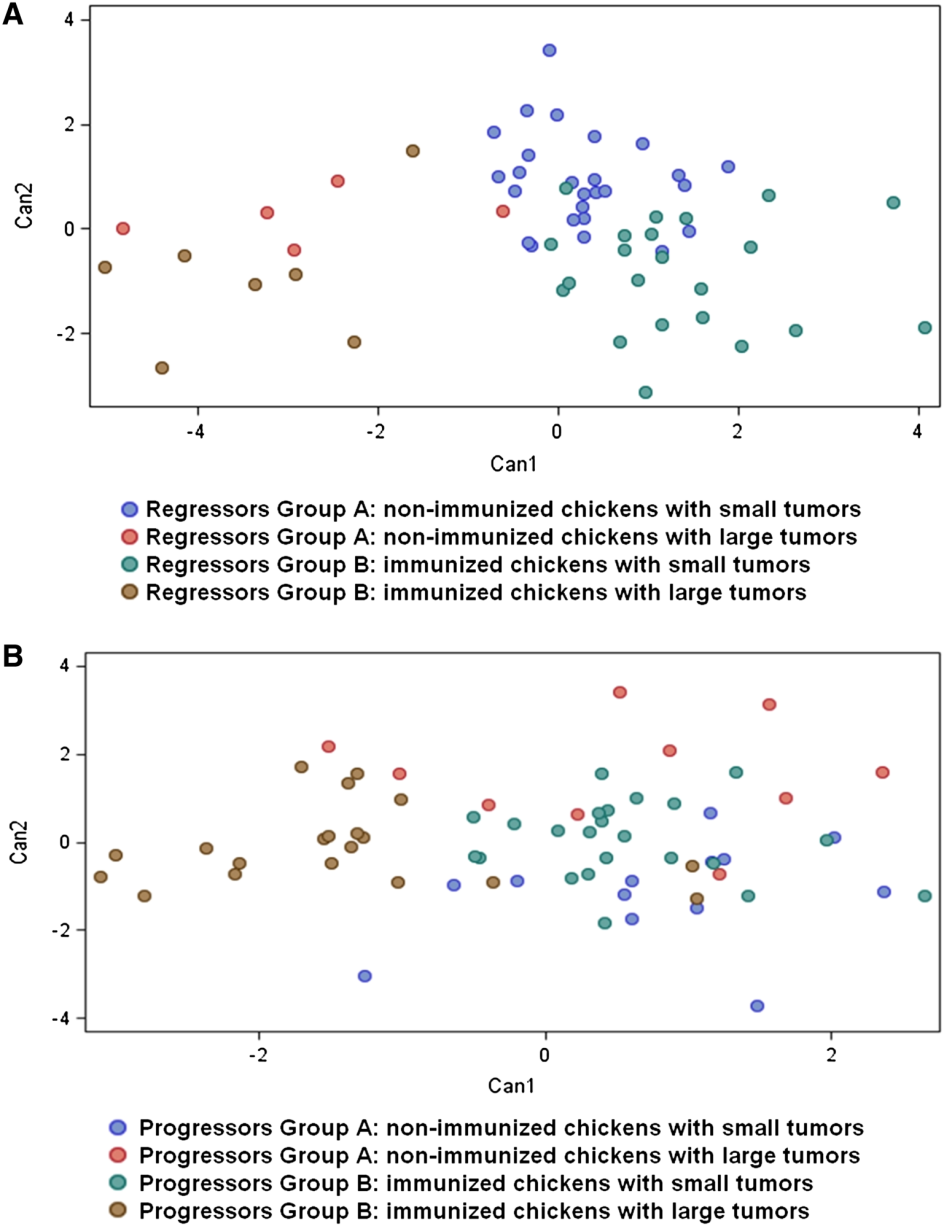
**Analysis of correlation of cytokine pattern with RSV-induced tumor status by real-time PCR.** Chickens were arbitrarily divided into two groups: small tumors—which stands for tumors up to 5 g (including completely regressed tumors) in the regressor lines CB and CB.R1 and for tumors up to 20 g in the progressor lines CC and CC.R1; large tumors—which stands for tumors above the weight 5 g in the CB and CB.R1 and above 20 g in the CC and CC.R1. **A** Classification of regressors individual responses by linear discriminant analysis. **B** Classification of progressors individual responses by linear discriminant analysis.

In the regressor lines, samples taken from chickens with small or large tumors formed distinct compact sets with significantly different expression cytokine profiles (Figure 7A). Analyses of progressor lines also showed significantly different expression profiles (Figure 7B). Among the set of 20 tested genes, 14 genes exhibited altered expression (Additional file 5). Fourteen genes (IL-1, IL-2, MIF, IL-4, IL-12, IL-18, IL-15, TLR7, TRAF5, XCL1, IL-17, TGF-β, iNOS, IFNγ) were found within the progressor lines, whereas six genes (IL-15, IL-18, IL-8, TGF-β, TLR7, iNOS) were found in the regressor lines. For detailed significance of data relations, see Table 1. The remaining marker genes (IFNα, IL-22, IL-13) were excluded from the panel as their expression did not change in response to immunization.

**Table 1:** **Significant differences in cytokine expression profiles between groups with small or large tumors of immunized and non-immunized chickens at particular time points**

	Imm.	Non-imm.		Imm.	Non-imm.
*CB, CB.RI*			*CC, CC.RI*		
14 dpc	+	Il-10*	14 dpc	–	IFNγ*
		Il-15**		Il-2**	–
		TLR*		Il-4**	Il-4*
28 dpc	Il-2**	–		Il-8**	–
	−	Il-4*		Il-12**	Il-12***
	Il-8**	–		–	Il-17*
	–	Il-12*		iNOS***	–
	Il-15***	Il-15**		XCL1*	XCL1*
	Il-18**	–		–	MIF***
	–	iNOS***		LITAF**	–
	–	XCL1**		TRAF5**	–
	–	MIF**	28 dpc	Il-1*	–
	TGF-β**	TGF-β *		Il-4*	Il-4*
	TLR7***	TLR7***		–	Il-12**
	TRAF5*	TRAF5**		–	Il-15***
				–	Il-17*
					XCL1***
				MIF**	–
				TGF-β*	–
				TLR7**	TLR7***
				LITAF**	–

For more detailed explanation, see Additional file 5.

^+^At this time point, no large tumor was developed.

* *p* < 0.05; ** *p* < 0.01; *** *p* < 0.001.

## Discussion

In this report, we used a new system of vaccination with antigen delivery into chicken APC. For the first time in the chicken, we verified the ability of fusion complexes of RSV antigens with SA to target specific biotinylated monoclonal antibody. For this purpose, we prepared a monoclonal antibody against chicken dendritic cell endocytic surface receptor CD205 (Ly75) and used it in the described antigen delivery system.

Using the newly prepared monoclonal anti chicken CD205 antibody, we observed chCD205 expression in different subsets of chicken leucocytes, mainly in antigen presenting cells as described by Vu Manh et al. [35]. Our observations were recently confirmed by Stainess et al. [4] who also found that CD205 expression was the highest in those chicken cells associated with antigen capture and presentation. We showed that the complex SA-RSV antigens-antibody was delivered specifically to the surface CD205 receptor and subsequently internalized. As pH in endosomes/lysosomes is between 4.5 and 5.5 [36], the FITC fluorescence signal was expected to decrease after receptor endocytosis and transfer of the complex into the cell endosome apparatus. This was confirmed by our internalization experiments where we showed decreased fluorescence signals after incubation of FITC-CD205-labeled chicken splenocytes at different temperatures.

In mammals, anti-CD205 antibodies are internalized via coated vesicles and are delivered to endosomal compartments [6]. The cytosolic domain of CD205 mediates efficient endocytosis and recycling through the late endosomes and MHCII-rich compartments [5]. In addition to its capacity to shuttle antigens from the extracellular space into specialized MHCII-rich lysosomal compartments, CD205 is also able to introduce antigens to the MHCI processing machinery.

We used poly I:C as an adjuvant, which acts as dsRNA recognized by TLR3 and thus mimics viral infection [37]. It is indicated that poly I:C can inhibit RSV transformation of chicken embryo fibroblasts in vitro [38], however, in our experiments we observed no such effect and, importantly, a rapid progression of tumors in susceptible lines CC and CC.R1 were not compromised at all. This distinction may be connected with e.g. in vivo use, different site of administration or a prolonged interval between immunization and challenge.

Antigen presentation by a subset of APC via MHCI molecules [39] provides a basis for protein vaccines when appropriately delivered. In this case, the genetic make-up of recipients, namely the ability of particular class I molecules to bind relevant peptides, is of great importance for successful protection. In our experiments, regressors, but not progressors, mount an effective CTL reaction against RSV tumor cell targets [13]. It turned out that this situation clearly corresponds to the presence of immunogenic peptide motifs within the RSV proteins that are stringently recognized by MHCI molecules (B-F12) of the regressor line, whereas only sparse relaxed motifs are available for MHCI molecules (B-F4) of the progressor line [17, 40]. A protective effect of vaccination with a single v-src-derived peptide identified to act against RSV challenge was demonstrated in the regressor chickens [41]. Many more peptides predicted to bind class I molecules from different disease-resistant chicken lines than from susceptible lines were found including Marek’s disease virus (MDV), infectious bursal disease virus (IBDV) and avian influenza virus [42–45]. It has been shown that some peptides derived from IBDV can be used as effective vaccine in chickens harboring the dominantly expressed class I molecule of the B12 haplotype. However, only one predicted peptide used for vaccination provided effective protection against the IBDV challenge, whereas vaccination with another peptide failed. In fact, even more severe signs of disease were observed in this case [43]. Thus, the data presented both in our paper and in other experimental systems strongly suggest the decisive role of the genetic make-up of recipients on the efficiency and final outcome of vaccination.

After activation of different chicken DC populations by direct in vivo antigen targeting of their surface receptor, we assume, in the case of regressors, antigen-dependent activation of both CD8^+^ cytotoxic T lymphocytes and effector CD4^+^ T cells, as well as activation of innate immunity cells. Other cell types such as natural killer cells (NK) and macrophages may also play a role in the immune responses against RSV tumors. In the case of progressors we do not suppose activation of an effective CTL reaction against RSV.

In this experiment, the cytokine response analyses showed significant up-regulation of IL-12, IL-2, IL-15 and IL-18 in the progressors, which reflects an anti-tumor immunity in the rapidly growing tumors. In this context, we can assume that DC at later stages of differentiation may regulate the activity of NK cells through the release of IL-12, IL-15 and IL-18, as has been reported [46]. DC are known to induce the growth and function of NK cells and play a crucial role in naïve T-cell priming. NK cells can influence the capability of DC to promote Th1 polarization, and the prevalence of IL-12, IL-2, IL-18, or IL-4 at inflammatory sites may differentially modulate the NK-cell interaction with DC [47]. Interestingly, we detected significant up-regulation of IL-17 in the group of immunized progressors. This is in accordance with the hypothesis that Th17 cells facilitate chemoattraction of Th1 cells [48].

In mammals, Cooper et al. [49] showed that IL-12-, IL-15- and IL-18-induced NK cells were memory-like cells and exhibited responses upon restimulation with cytokines (IL-2 and IL-15). DC rapidly stimulate NK cells through production of cytokines, such as IL-2, IL-12, IL-15 and IL-18. It has been reported that DC vaccination generates long-term cell-based resistance against tumor cells in an antigen-independent manner [49]. In the process of oncogenesis, we observed intriguing down-regulation of LITAF mainly in the group of immunized progressors with large tumors. Simultaneously, we did not detect any significant increase in the expression of IFNγ mainly in the immunized groups of progressors, which would indicate involvement of NK cells in the tumor interactions.

In chickens, there are several C-type lectins that are similar to certain mammalian natural killer receptor genes located in the close vicinity of or even within the MHC-B [50–54]. Other novel families of potential chicken Ig-like receptors were also discovered [55]. B-NK was found on several T-cell subsets and also on CD3^−^CD8^+^ sorted splenocytes that were in vitro expanded by IL-2 and on embryonic splenocytes, both of which resemble chicken NK cells [56]. Our findings of specifically activated cytokines known to act in the NK-cell pathway point to their importance in our experimental system of MHC-B congenic lines of chickens, in addition to the previously described Th1 antitumor response. NK cell receptors could be considered as both activating and inhibitory and the balance of their effects determines the final character of the immune response. It is within the context of these subtle and diverse factors, that the data presented in this report must be considered. We performed in vitro restimulation of PBMC where we evaluated supernatant levels of three cytokines (IL-2, IL-12 and IFNγ). Unfortunately, the availability of specific antibodies against chicken cytokines is limited; other cytokines were not evaluated therefore. All cytokine levels were significantly increased in the immunized groups restimulated with v-src antigen. This suggests that the direct delivery of the antigens via CD205 receptor may also induce the classical Th1 immune profile.

Interestingly, both congenic lines sharing the B-G4 allele, CB.R1 (regressors) and CC (progressors), showed a rather adverse effect of vaccination (for the composition of the MHC-B haplotypes see the “Materials and methods”). The true function of the B-G genes—known as MHC-B class IV—is still elusive, namely with respect to their immunological functions [26]. The significant role of the B-G genes in the relative resistance to MDV-induced lymphoma has also been described in another experimental system of MHC-B recombinant chickens [28]. Our results suggest that the genotype in the B-G genes (MHC—class IV) correlates to some extent in the response to the vaccination despite the general status of regressors or progressors governed primarily by the B-F (MHCI) genes. So far, we can only speculate what putative suppressive signal of the B-G4 molecule might influence the significant aggravating of the vaccination response in our model system.

We report in this study the first description of a novel system of vaccination of chickens based on a novel tool for antigen delivery into chicken DC. A novel anti-chCD205 monoclonal antibody has been developed and the internalization of the antigen through this receptor has been confirmed. Cytokine expression profile differences in RSV-challenged progressor and regressor chicken lines have been described. Based on the increased expression of cytokines specific for activated DC (IL-12, IL-15 and IL-18) we have shown partial stimulation of specific cell types involved in cell-mediated immunity, although the only significant effect of immunization on the tumor size has been confirmed in IL-12. Further investigation is needed to elucidate the apparently complex mechanism of the response to vaccination and to optimize the system of antigen delivery to avoid unwanted suppressive effects.

## Additional files

**Additional file 1.** **Supplementary Materials and methods**. Additional description of anti-chicken CD205 monoclonal antibody preparation, the construction, expression and purification of the recombinant SA-RSV fusion proteins and the description of amino acid sequence CD205 protein.

**Additional file 2.** **List of primers used in the study.** Sequences of primers used in anti-CD205 antigen cloning.

**Additional file 3.** **List of primers used in the study.** Sequences of primers used in anti-CD205 antigen cloning.

**Additional file 4.** **List of primers used for RT-PCR in this study.** Sequences of primers used in cytokine pattern analyses, primers length, amplicon size and amplified genes access PubMed database numbers.

**Additional file 5.** **Cytokine profiles of selected genes in progressor and regressor groups of immunized and non-immunized chickens.** These data show the expression profiles of all observed cytokines in progressor and regressor groups of immunized and non-immunized chickens. The expression value after challenge was normalized to the basic value of expression in the day of challenge.

## Abbreviations

APC: antigen-presenting cells
DC: dendritic cells
dpc: days post challenge
FFU: focus-forming units
MHC: major histocompatibility complex
PBMC: peripheral blood mononuclear cells
NK: natural killer cells
PCA: principal component analysis
RSV: Rous sarcoma virus
RT-PCR: real-time polymerase chain reaction
SA: streptavidin
sc: subcutaneously

## References

[1] Gajewski TF, Schreiber H, Fu Y-X (2013). Innate and adaptive immune cells in the tumor microenvironment. Nat Immunol.

[2] Gabrilovich DI, Ostrand-Rosenberg S, Bronte V (2012). Coordinated regulation of myeloid cells by tumours. Nat Rev Immunol.

[3] Burkholder B, Huang R-Y, Burgess R, Luo S, Jones VS, Zhang W, Lv Z-Q, Gao C-Y, Wang B-L, Zhang Y-M, Huang R-P (2014). Tumor-induced perturbations of cytokines and immune cell networks. Biochim Biophys Acta.

[4] Staines K, Young JR, Butter C (2013). Expression of chicken DEC205 reflects the unique structure and function of the avian immune system. PLoS One.

[5] Jiang W, Swiggard WJ, Heufler C, Peng M, Mirza A, Steinman RM, Nussenzweig MC (1995). The receptor DEC-205 expressed by dendritic cells and thymic epithelial cells is involved in antigen processing. Nature.

[6] Bonifaz L, Bonnyay D, Mahnke K, Rivera M, Nussenzweig MC, Steinman RM (2002). Efficient targeting of protein antigen to the dendritic cell receptor DEC-205 in the steady state leads to antigen presentation on major histocompatibility complex class I products and peripheral CD8 + T cell tolerance. J Exp Med.

[7] Trumpfheller C, Finke JS, López CB, Moran TM, Moltedo B, Soares H, Huang Y, Schlesinger SJ, Park CG, Nussenzweig MC, Granelli-Piperno A, Steinman RM (2006). Intensified and protective CD4 + T cell immunity in mice with anti-dendritic cell HIV gag fusion antibody vaccine. J Exp Med.

[8] Steinman RM (2008). Dendritic cells in vivo: a key target for a new vaccine science. Immunity.

[9] Stanek O, Linhartova I, Majlessi L, Leclerc C, Sebo P (2012). Complexes of streptavidin-fused antigens with biotinylated antibodies targeting receptors on dendritic cell surface: a novel tool for induction of specific T-cell immune responses. Mol Biotechnol.

[10] Dong H, Stanek O, Salvador FR, Länger U, Morillon E, Ung C, Sebo P, Leclerc C, Majlessi L (2013). Induction of protective immunity against Mycobacterium tuberculosis by delivery of ESX antigens into airway dendritic cells. Mucosal Immunol.

[11] Brugge JS, Erikson RL (1977). Identification of a transformation-specific antigen induced by an avian sarcoma virus. Nature.

[12] Plachy J, Pink JR, Hála K (1992). Biology of the chicken MHC (B complex). Crit Rev Immunol.

[13] Plachý J, Hála K, Hejnar J, Geryk J, Svoboda J (1994). src-specific immunity in inbred chickens bearing v-src DNA- and RSV-induced tumors. Immunogenetics.

[14] Plachý JV, Hejnar JV, Trtková K, Trejbalová K, Svoboda J, Hála K (2001). DNA vaccination against v-src oncogene-induced tumours in congenic chickens. Vaccine.

[15] Taylor RL, Ewert DL, England JM, Halpern MS (1992). Major histocompatibility (B) complex control of the growth pattern of v-src DNA-induced primary tumors. Virology.

[16] Gelman IH, Hanafusa H (1993). src-specific immune regression of Rous sarcoma virus-induced tumors. Cancer Res.

[17] Kaufman J, Völk H, Wallny HJ (1995). A “minimal essential Mhc” and an “unrecognized Mhc”: two extremes in selection for polymorphism. Immunol Rev.

[18] Kaufman J, Milne S, Göbel TW, Walker BA, Jacob JP, Auffray C, Zoorob R, Beck S (1999). The chicken B locus is a minimal essential major histocompatibility complex. Nature.

[19] Wallny H-J, Avila D, Hunt LG, Powell TJ, Riegert P, Salomonsen J, Skjødt K, Vainio O, Vilbois F, Wiles MV, Kaufman J (2006). Peptide motifs of the single dominantly expressed class I molecule explain the striking MHC-determined response to Rous sarcoma virus in chickens. Proc Natl Acad Sci U S A.

[20] Walker BA, Hunt LG, Sowa AK, Skjødt K, Göbel TW, Lehner PJ, Kaufman J (2011). The dominantly expressed class I molecule of the chicken MHC is explained by coevolution with the polymorphic peptide transporter (TAP) genes. Proc Natl Acad Sci U S A.

[21] Zhang L, Katselis GS, Moore RE, Lekpor K, Goto RM, Hunt HD, Lee TD, Miller MM (2012). MHC class I target recognition, immunophenotypes and proteomic profiles of natural killer cells within the spleens of day-14 chick embryos. Dev Comp Immunol.

[22] Hofmann M, Nussbaum AK, Emmerich NP, Stoltze L, Schild H (2001). Mechanisms of MHC class I-restricted antigen presentation. Expert Opin Ther Targets.

[23] Salomonsen J, Chattaway JA, Chan ACY, Parker A, Huguet S, Marston DA, Rogers SL, Wu Z, Smith AL, Staines K, Butter C, Riegert P, Vainio O, Nielsen L, Kaspers B, Griffin DK, Yang F, Zoorob R, Guillemot F, Auffray C, Beck S, Skjødt K, Kaufman J (2014). Sequence of a complete chicken BG haplotype shows dynamic expansion and contraction of two gene lineages with particular expression patterns. PLoS Genet.

[24] Plachý J (1984). Hierarchy of the B (MHC) haplotypes controlling resistance to rous sarcomas in a model of inbred lines of chickens. Folia Biol (Praha).

[25] Plachý J (1988). The B-G region genes of the chicken MHC are responsible for lethal graft-versus-host disease in newly hatched chickens. Folia Biol (Praha).

[26] Kaufman J, Skjødt K, Salomonsen J (1991). The B-G multigene family of the chicken major histocompatibility complex. Crit Rev Immunol.

[27] Salomonsen J, Eriksson H, Skjødt K, Lundgreen T, Simonsen M, Kaufman J (1991). The “adjuvant effect” of the polymorphic B-G antigens of the chicken major histocompatibility complex analyzed using purified molecules incorporated in liposomes. Eur J Immunol.

[28] Goto RM, Wang Y, Taylor RL, Wakenell PS, Hosomichi K, Shiina T, Blackmore CS, Briles WE, Miller MM (2009). BG1 has a major role in MHC-linked resistance to malignant lymphoma in the chicken. Proc Natl Acad Sci U S A.

[29] Plachý J, Vilhelmová M (1984). Syngeneic lines of chickens. VII. The lines derived from the recombinants at the B complex (MHC) of Rous sarcoma regressor and progressor inbred lines of chickens. Folia Biol (Praha).

[30] Méric C, Spahr PF (1986). Rous sarcoma virus nucleic acid-binding protein p12 is necessary for viral 70S RNA dimer formation and packaging. J Virol.

[31] Hong YH, Lillehoj HS, Lillehoj EP, Lee SH (2006). Changes in immune-related gene expression and intestinal lymphocyte subpopulations following *Eimeria maxima* infection of chickens. Vet Immunol Immunopathol.

[32] Rothwell L, Young JR, Zoorob R, Whittaker CA, Hesketh P, Archer A, Smith AL, Kaiser P (2004). Cloning and characterization of chicken IL-10 and its role in the immune response to *Eimeria maxima*. J Immunol.

[33] Jolliffe IT (2002). Principal Component Analysis.

[34] Martin MM, Lindqvist L (1975). The pH dependence of fluorescein fluorescence. J Lumin.

[35] Vu Manh T-P, Marty H, Sibille P, Le Vern Y, Kaspers B, Dalod M, Schwartz-Cornil I, Quéré P (2014). Existence of conventional dendritic cells in *Gallus gallus* revealed by comparative gene expression profiling. J Immunol.

[36] Sorkin A, Von Zastrow M (2002). Signal transduction and endocytosis: close encounters of many kinds. Nat Rev Mol Cell Biol.

[37] Martins KAO, Bavari S, Salazar AM (2015). Vaccine adjuvant uses of poly-IC and derivatives. Expert Rev Vaccines.

[38] Dodge WH, Moscovici C (1972). Effect of poly I: C on transformation by Rous sarcoma virus. Proc Soc Exp Biol Med.

[39] Rock KL (1996). A new foreign policy: MHC class I molecules monitor the outside world. Immunol Today.

[40] Kaufman J, Wallny HJ (1996). Chicken MHC molecules, disease resistance and the evolutionary origin of birds. Curr Top Microbiol Immunol.

[41] Hofmann A, Plachy J, Hunt L, Kaufman J, Hala K (2003). v-src oncogene-specific carboxy-terminal peptide is immunoprotective against Rous sarcoma growth in chickens with MHC class I allele B-F12. Vaccine.

[42] Koch M, Camp S, Collen T, Avila D, Salomonsen J, Wallny HJ, van Hateren A, Hunt L, Jacob JP, Johnston F, Marston DA, Shaw I, Dunbar PR, Cerundolo V, Jones EY, Kaufman J (2007). Structures of an MHC class I molecule from B21 chickens illustrate promiscuous peptide binding. Immunity.

[43] Butter C, Staines K, van Hateren A, Davison TF, Kaufman J (2013). The peptide motif of the single dominantly expressed class I molecule of the chicken MHC can explain the response to a molecular defined vaccine of infectious bursal disease virus (IBDV). Immunogenetics.

[44] Hou Y, Guo Y, Wu C, Shen N, Jiang Y, Wang J (2012). Prediction and identification of T cell epitopes in the H5N1 influenza virus nucleoprotein in chicken. PLoS One.

[45] Reemers SS, van Haarlem DA, Sijts AJ, Vervelde L, Jansen CA (2012). Identification of novel avian influenza virus derived CD8 + T-cell epitopes. PLoS One.

[46] Andrews DM, Andoniou CE, Scalzo AA, van Dommelen SLH, Wallace ME, Smyth MJ, Degli-Esposti MA (2005). Cross-talk between dendritic cells and natural killer cells in viral infection. Mol Immunol.

[47] Agaugué S, Marcenaro E, Ferranti B, Moretta L, Moretta A (2008). Human natural killer cells exposed to IL-2, IL-12, IL-18, or IL-4 differently modulate priming of naive T cells by monocyte-derived dendritic cells. Blood.

[48] Khader SA, Bell GK, Pearl JE, Fountain JJ, Rangel-Moreno J, Cilley GE, Shen F, Eaton SM, Gaffen SL, Swain SL, Locksley RM, Haynes L, Randall TD, Cooper AM (2007). IL-23 and IL-17 in the establishment of protective pulmonary CD4 + T cell responses after vaccination and during *Mycobacterium tuberculosis* challenge. Nat Immunol.

[49] Cooper MA, Elliott JM, Keyel PA, Yang L, Carrero JA, Yokoyama WM (2009). Cytokine-induced memory-like natural killer cells. Proc Natl Acad Sci U S A.

[50] Kaufman J (1999). Co-evolving genes in MHC haplotypes: the “rule” for nonmammalian vertebrates?. Immunogenetics.

[51] Rogers SL, Göbel TW, Viertlboeck BC, Milne S, Beck S, Kaufman J (2005). Characterization of the chicken C-type lectin-like receptors B-NK and B-lec suggests that the NK complex and the MHC share a common ancestral region. J Immunol.

[52] Rogers SL, Kaufman J (2008). High allelic polymorphism, moderate sequence diversity and diversifying selection for B-NK but not B-lec, the pair of lectin-like receptor genes in the chicken MHC. Immunogenetics.

[53] Shiina T, Briles WE, Goto RM, Hosomichi K, Yanagiya K, Shimizu S, Inoko H, Miller MM (2007). Extended gene map reveals tripartite motif, C-type lectin, and Ig superfamily type genes within a subregion of the chicken MHC-B affecting infectious disease. J Immunol.

[54] Rogers S, Shaw I, Ross N, Nair V, Rothwell L, Kaufman J, Kaiser P (2003). Analysis of part of the chicken Rfp-Y region reveals two novel lectin genes, the first complete genomic sequence of a class I alpha-chain gene, a truncated class II beta-chain gene, and a large CR1 repeat. Immunogenetics.

[55] Straub C, Neulen M-L, Sperling B, Windau K, Zechmann M, Jansen CA, Viertlboeck BC, Göbel TW (2013). Chicken NK cell receptors. Dev Comp Immunol.

[56] Viertlboeck BC, Wortmann A, Schmitt R, Plachý J, Göbel TW (2008). Chicken C-type lectin-like receptor B-NK, expressed on NK and T cell subsets, binds to a ligand on activated splenocytes. Mol Immunol.

