# Supplementary material for: Cytokine response to the RSV antigen delivered by dendritic cell-directed vaccination in congenic chicken lines
Source: Vet Res. 2017 Apr 5;48:18. doi: 10.1186/s13567-017-0423-8 (PMC5382389; doi:10.1186/s13567-017-0423-8)
Supplement: Supplementary file 1 — Additional file 1. Supplementary Materials and methods. Additional description of anti-chicken CD205 monoclonal antibody preparation, the construction, expression and purification of the recombinant SA-RSV fusion proteins and the description of amino acid sequence CD205 protein. [file 13567_2017_423_MOESM1_ESM.docx]

**Additional file 5 Materials and methods**

**Anti-chicken Ly75 monoclonal antibody preparation**

The amino acid (aa) sequence of the chicken lymphocyte antigen 75 (chLy75) precursor (NP_001032925.1) was analyzed according to its position in the plasma membrane (Phobius predictor, [57]). The extracellular domain of this receptor was divided into six fragments (A-F). These fragments were PCR amplified (Additional file 2) using the templates of cDNA of domestic chicken (*Gallus gallus*) and inserted into the restored NcoI and XhoI sites of the pET28b expression vector (Novagen, Germany). The parts of the recombinant Ly75 receptor were produced in *E. coli* Rosetta 2 (DE3) cells (Novagen, Germany), extracted from inclusion bodies with a buffer containing 8 M urea, and isolated by anion-exchange chromatography on DEAE Sepharose. For production of monoclonal antibodies we used only parts B (aa 227-485) and F (aa 1406-1668) of the extracellular domain of chicken lymphocyte antigen 75 precursor, because the expression yield of fragments A, C and E was low, and fragment D could not be purified.

BALB/c mice (6-week-old) were immunized intraperitoneally with 100 µg of purified chLy75 (B+F) combined with Freund’s adjuvant (Sigma, St. Louis, MO, USA). Spleen lymphocytes from immunized mice were fused with non-secreting mouse myeloma SP2 cells three days after the final boost, resulting in 365 hybridomas that produced mAbs against chLy75 (B+F). Hybridomas were selected in medium supplemented with HAT (Sigma). The samples of hybridoma supernatants were screened by ELISA and flow cytometry using leukocytes prepared from chicken spleen. mAbs with high binding specificity for recombinant chLy75 were identified by flow cytometry, and one of them was selected for further characterization and biotin conjugation (Apronex, Prague, Czech Republic).

**Construction, expression and purification of the recombinant SA-RSV fusion proteins**

The construction and purification of the SA-Ag fusion protein was described recently [9, 10]. Briefly, the open reading frames encoding RSV antigens genes v-src, pol, and gag were PCR-amplified with specific primers (Additional file 3) using the template of pAPrC plasmid DNA containing a complete, non-permutated molecular clone of C subgroup RSV Prague strain [58], and fused in frame to the 3’ end of a codon-optimized synthetic gene encoding residues 13-139 of SA from *Streptomyces avidinii*. The antigen v-src was divided into two overlapping parts – including aa 4-285 (V-srcA) and aa 241-526 (V-srcB) – because of its size. The genes for gag and pol antigens were genetically attached to the 3´terminal part of the SA gene in the full-length sequence. For ENV antigen, we obtained the synthetic codon-optimized gene (GenScript, USA). The env antigen was divided into two overlapping parts – ENVoep (19-26; 65-304 aa) and ENVly (279-506 aa). The SA-ENV constructs did not include the signal and the transmembrane domains – 1-56 and 507-603 aa, respectively. Only the hypothetical epitope “KDSKEKPL” of aa 19-26 was added to the SA-ENVoep fusion protein (Figure 1A). Sequence-confirmed SA-RSV fusion proteins were produced as soluble tetramers in *E. coli* Artic Express DE3 cells (Stratagene, CA, USA) transformed with pET28b-SA-RSV expression plasmid as described [10]. The tetramers of the fusion protein were extracted from the cytosol (cells debris) with 2 M urea in 50 mM ammonium acetate buffered to pH 9 by 25% NH3xH2O (AC buffer) and purified by affinity chromatography on 2-Iminobiotin-Agarose (Sigma-Aldrich, MO, USA), with two forms of fusion proteins finally separated. The lipopolysaccharide content was reduced by passage through EndoTrap columns (Profos, Germany) to levels below 50 endotoxin units of lipopolysaccharide per mg of protein. The purity of the SA-RSV fusion proteins was checked in Tris-Tricin SDS PAGE gel (Figure 2B).

**CD205 receptor**

MGRPAGRCAAACCIAWLLACCAARRDDTGSGAF***TIRHDTLNKCIQVKNSRIVVDDCKETSEALWKWVSQSRLFHLGTKQCLGLDIFTKLPSRLRMVDCNSDLRLWWRCADGSVVGASQYKVTVRSAYVTASINASDQWRSNN***SSADICQYPYHEIYTKDGNSYGKPCEFPFLYNMTWHHDCIQDGTHTGRKWCATSEDYTRDGKWGICLQPEDGCHDIWEHDASSQ***HCYQFNTQSALSWKEAYVSCQRQGGDLLSIQDASELNYIQAKDDIAEIFWIGLNQLDVSRGWQWSDHKPLNFVNWHPDMWDLSPLDGTSCVAMNAASGQWRSYHCGNPLPYVCKKSFKEVSNLTEFWRHVNTRCDAGWLPHNGFCYMLIHNQASWSTADQLCKANKSNLISIHSLADVELIVTKLHNDAREEVWVGLRNEDVPTLFKWSDRTDVVFTYWDQNEPSVPFNATPNCVSYSGKLGQWRVKSCEENLKYVCKK***KGKILNETKSDKNCSLDEGWEMHGN***YCYKILNTEVSFGAECNLTVTNRFEQEFINSLIRKHTKVEEKYFWTGLQDISQSGMYSWAAVDGEKSEAVTYTNWNSLQPEFSGGCVAMSSGRSLGKWETKDCKTTKAFPVCKKYIGLPKEPEVLPKPSDPCPPGWHNGSGLACYKFFHSERVLRTRTWEEAERFCEALGGHLPSFTHTEEIKTLHSILRKIISNDRWVWVGMNKRSPDSLGTWQWSDDKPVTSLVLPHDYLEDEYDTRDCVALKTFQFSRRSFWRFYFHEGRDLEFYFKPFDCEAKLEWVCQI***TKGSTPKTPEWYIPDEIGIHGVPLVVDGAEL***WFVPDKNVSFQEAISYCQKNDSELASVESYPKLRTILSQIEKLSNSEQKWWLKFIDYGYSYHSPLQLFPRFHDRSLRDCWYVSRKSWYRDYPVNCNMKLPFICEKNNASLLEKHDPSYRPVTGGCPKGWLRFRNKCFLKMKSEYLTFNAANEKCVTFGGSLPCISSQAEQDFITSLLPQMPRDIWIGLQFLFSTRENKWIDESRLLYSNFHPLLTGRLRKIPLDLFDEEFNNQCGVILNDPKSHYVGTWNFTACADRHFLGICQR***PIGIGAADNQTEQVLNDTFSYHNVQ***YKLILNNLTWNDAMAACIGQKMQLVSITDQFQQASLAVQAALHDYPLWIGLFSRDGGKHYGWLDGKHVSFSRWSEDDEETSEECVYLDTDGFWKTSDCYSENRGAICYSSEKKDEKEQVTQVKCPHKIKNTPWISFRNNCYTFMITKNRWREMKSQEAHHLCKKMNPEAFVLSVRDEEENNFVAEQLHSFSGLAVWVWLGVIYDDSDKVLKWYDETYLTYNNWRLGRPIIKKNSFFAGVNLDGFWDIYNYSQSWQAH***HYNVYSILACKIERGPQQHKPPLPEFIPHGDVT***YRILQKKLTWYDAVRECKQNMSDLASVHSESQQLFLEDIVKQDGYSLWLGLSIHDGSKANFEWSDGSSFDYYPWELENSNTTENCVLLDTKGSWNRAKCTNVAEGAICYSFSNKKQLEQKQVSRASGCSQLSGELPWIQYKDHCYAFDMAFYNFSVYNVEDAKKVCKKLNPSAALLTIGDAEENAFVSAHIKKNDLITRKVWLGLTQSSTGQTLHWLDGSSVNYANWDNRTTELSEKCSVITSTTGKWSKVDCSRSQSRVVCK***APLGSNHTGVAVAFALLVILVLVLGLVWFICKKKRLHWSAFSSVHYQRGLNDDEADDVFTKDGY

ly75A – 34-142

**ly75B – 227-485**

ly75C – 511-794

ly75D – 826-1090

ly75E – 1116-1372

**ly75F – 1406-1668**

The amino acid sequence of the lymphocyte antigen 75 precursor (NCBI Reference Sequence: NP_001032925.1) was analyzed according to its position in the plasma membrane (Phobius predictor, [57]). The bold highlighted parts were chosen for recombinant protein preparation. Finally, only the bold and underlined highlighted parts were prepared as the recombinant proteins and used for preparation of monoclonal antibodies.
