# Supplementary material for: Cytokine response to the RSV antigen delivered by dendritic cell-directed vaccination in congenic chicken lines
Source: Vet Res. 2017 Apr 5;48:18. doi: 10.1186/s13567-017-0423-8 (PMC5382389; doi:10.1186/s13567-017-0423-8)
Supplement: Supplementary file 2 — Additional file 2. List of primers used in the study. Sequences of primers used in anti-CD205 antigen cloning. [file 13567_2017_423_MOESM2_ESM.docx]

**Additional file 2 PCR primers used in cloning.**

| primer name | sequence 5’→3’ |
| --- | --- |
| vsrcAfor | TACCATGGATACTAGTGGGAGCAGCAAGAGCAAGC |
| vsrcArev | ATAAGCTTGAATTCGGTCCCCATCCAGA |
| vsrcBfor | TACCATGGTAACTAGTCTGGCCAACGTCTGCCC |
| vsrcBrev | GCAAGCTTTTACTCAGCGACCTCCAAC |
| forPOL | TAGGATCCACTAGTCCTGTGTGGATTGACCAGT |
| revPOL | TACTCGAGTTAGAATTCTAACTTGTACCCAAGATATT |
| forGAG | TAGGATCCACTAGTCCAAAATTGATCACAAGACT |
| revGAG | TACTCGAGTTAGAATTCCCGTATAAGCTGCTGAATAT |

Sequences of primers used in cloning of RSV genes.
