# Supplementary material for: Cytokine response to the RSV antigen delivered by dendritic cell-directed vaccination in congenic chicken lines
Source: Vet Res. 2017 Apr 5;48:18. doi: 10.1186/s13567-017-0423-8 (PMC5382389; doi:10.1186/s13567-017-0423-8)
Supplement: Supplementary file 3 — Additional file 3. List of primers used in the study. Sequences of primers used in anti-CD205 antigen cloning. [file 13567_2017_423_MOESM3_ESM.docx]

**Additional file 3 Primers used for PCR amplification of six parts of Ly75.**

| **Fragment** |  | **Oligonucleotide sequence (5’→3’)** |
| --- | --- | --- |
| **ly75A** | ly75Afor | TA CCA TGG AT ACC ATC AGG CAC GAC AC |
|  | ly75Arew | TA CTC GAG GTT GTT GCT TCT CCA CTG G |
| **ly75B** | ly75Bfor | TA CCA TGG AT CAT TGC TAC CAG TTC AAC AC |
|  | ly75Brew | TA CTC GAG TTT CTT ACA CAC GTA TTT CAA G |
| **ly75C** | ly75Cfor | TA CCA TGG AT TAC TGC TAC AAG ATT TTG AAC |
|  | ly75Crew | TA CTC GAG TAT CTG GCA GAC CCA TTC A |
| **ly75D** | ly75Dfor | TA CCA TGG AT TGG TTT GTA CCA GAT AAA AAC G |
|  | ly75Drew | TA CTC GAG ACG CTG GCA TAT ACC CA |
| **ly75E** | ly75Efor | TA CCA TGG AT TAT AAA CTA ATC CTG AAC AAT C |
|  | ly75Erew | TA CTC GAG ATG AGC TTG CCA ACT TTG |
| **ly75F** | ly75Ffor | TA CCA TGG AT TAC AGA ATT CTT CAG AAA AAG T |
|  | ly75Frew | TA CTC GAG TTT ACA AAC CAC TCT GCT TTG |
