# Supplementary material for: Cytokine response to the RSV antigen delivered by dendritic cell-directed vaccination in congenic chicken lines
Source: Vet Res. 2017 Apr 5;48:18. doi: 10.1186/s13567-017-0423-8 (PMC5382389; doi:10.1186/s13567-017-0423-8)
Supplement: Supplementary file 4 — Additional file 4. List of primers used for RT-PCR in this study. Sequences of primers used in cytokine pattern analyses, primers length, amplicon size and amplified genes access PubMed database numbers. [file 13567_2017_423_MOESM4_ESM.docx]

**Additional file 4 Sequences of used primers.**

| Primer name | Sequence | Length | Product | Access no. |
| --- | --- | --- | --- | --- |
| IL1B F | GTG AGG CTC AAC ATT GCG CTG TA | 23 | 301 | FJ537840 |
| IL1B R | TGT CCA GGC GGT AGA AGA TGA AG | 23 |  |  |
| IL2 F | TCT GGG ACC ACT GTA TGC TCT | 21 | 256 | AJ224516^*^ |
| IL2 R | ACA CCA GTG GGA AAC AGT ATC A | 22 |  |  |
| IL4 F | AAC ATG CGT CAG CTC CTG AAT | 21 | 98 | NM_001007079^**^ |
| IL4 R | TCT GCT AGG AAC TTC TCC ATT GAA | 24 |  |  |
| IL8 F | GGC TTG CTA GGG GAA ATG A | 19 | 200 | FJ907874^*^ |
| IL8 R | AGC TGA CTC TGA CTA GGA AAC TGT | 24 |  |  |
| IL10 F | CAT GCT GCT GGG CCT GAA | 18 | 94 | AJ621254^**^ |
| IL10 R | CGT CTC CTT GAT CTG CTT GAT G | 22 |  |  |
| IL12 F | TGG TCC ACG CTT TGC AGA T | 19 | 140 | AJ564201 |
| IL12 R | AAG GTT AAG GCG TGG CTT CTT A | 22 |  |  |
| IL13 F | CTG CCC TTG CTC TCC TCT GT | 20 | 123 | AJ621250 |
| IL13 R | CCT GCA CTC CTC TGT TGA GCT T | 22 |  |  |
| IL15 F | TCT GTT CTT CTG TTC TGA GTG AT | 23 | 243 | NM_204571^*^ |
| IL15 R | AGT GAT TTG CTT CTG TCT TTG GT | 23 |  |  |
| IL17 F | TAT CAG CAA ACG CTC ACT GG | 20 | 110 | AJ493595 |
| IL17 R | AGT TCA CGC ACC TGG AAT G | 19 |  |  |
| IL18 F | GGA ATG CGA TGC CTT TTG | 18 | 264 | NM_204608^*^ |
| IL18 R | ATT TTC CCA TGC TCT TTC TCA | 21 |  |  |
| IL22 F | CAG ACT CAT CGG TCA GCA AA | 20 | 122 | AJ617782 |
| IL22 R | GGT ACC TCT CCT TGG CCT CT | 20 |  |  |
| IFN α F | GAC ATC CTT CAG CAT CTC TTC A | 22 | 238 | AM049251^*^ |
| IFN α R | AGG CGC TGT AAT CGT TGT CT | 20 |  |  |
| INF γ F | ACA CTG ACA AGT CAA AGC CGC | 21 | 129 | NM_205149 |
| INF γ R | AGT CGT TCA TCG GGA GCT TG | 20 |  |  |
| iNOS F | TGG GTG GAA GCC GAA ATA | 18 | 241 | U34045^*^ |
| iNOS R | GTA CCA GCC GTT GAA AGG AC | 20 |  |  |
| TNF α F | AAT TTG CAG GCT GTT TCT GC | 20 | 112 | AY765397 |
| TNF α R | TAT GAA GGT GGT GCA GAT GG | 20 |  |  |
| TLR7 F | TCA GAG GTG GCT GCA CAC | 18 | 60 | NM_001011688 |
| TLR7 R | CAA CAG TGC ATT TGA CGT CCT T | 22 |  |  |
| MIF F | GCA GCC TCT ACA GCA TTG G | 19 | 229 | NM_001305091^*^ |
| MIF R | TCT AAC GGG CAG CAC GAG | 18 |  |  |
| LT F | GGA TTT AAG GGT GAA CAG TAG ATG | 24 | 254 | NM_205046^*^ |
| LT R | TAG AAA TAG AAA GCC CGA GGA T | 22 |  |  |
| TRAF5 F | TGA TTA TCC CAT GCC TTG TCT | 21 | 283 | NM_204219^*^ |
| TRAF5 R | CTC TGC TAG CTG CTG GAT TTT A | 22 |  |  |
| TGFβ F | CGG GAC GGA TGA GAA GAA C | 19 | 258 | JQ423909^*^ |
| TGFβ R | CGG CCC ACG TAG TAA ATG AT | 20 |  |  |
| 28S F | GGC GAA GCC AGA GGA AAC | 18 | 62 | DQ018756^**^ |
| 28S R | GAC GAC CGA TTT GCA CGT C | 19 |  |  |
| GAPDH F | CAT CGT GCA CCA CCA ACT G | 19 | 179 | NM_204305 |
| GAPDH R | CGC TGG GAT GAT GTT CTG G | 19 |  |  |

Sequences of primers used in cytokine pattern analyses, primers length, amplicon size and amplified genes access PubMed database numbers.

^*^ These primers were described by [55]; ^**^ these primers were described by [56].
