# Supplementary figures and images for: Cytokine response to the RSV antigen delivered by dendritic cell-directed vaccination in congenic chicken lines
Source: Vet Res. 2017 Apr 5;48:18. doi: 10.1186/s13567-017-0423-8 (PMC5382389; doi:10.1186/s13567-017-0423-8)

# IFN $\gamma$

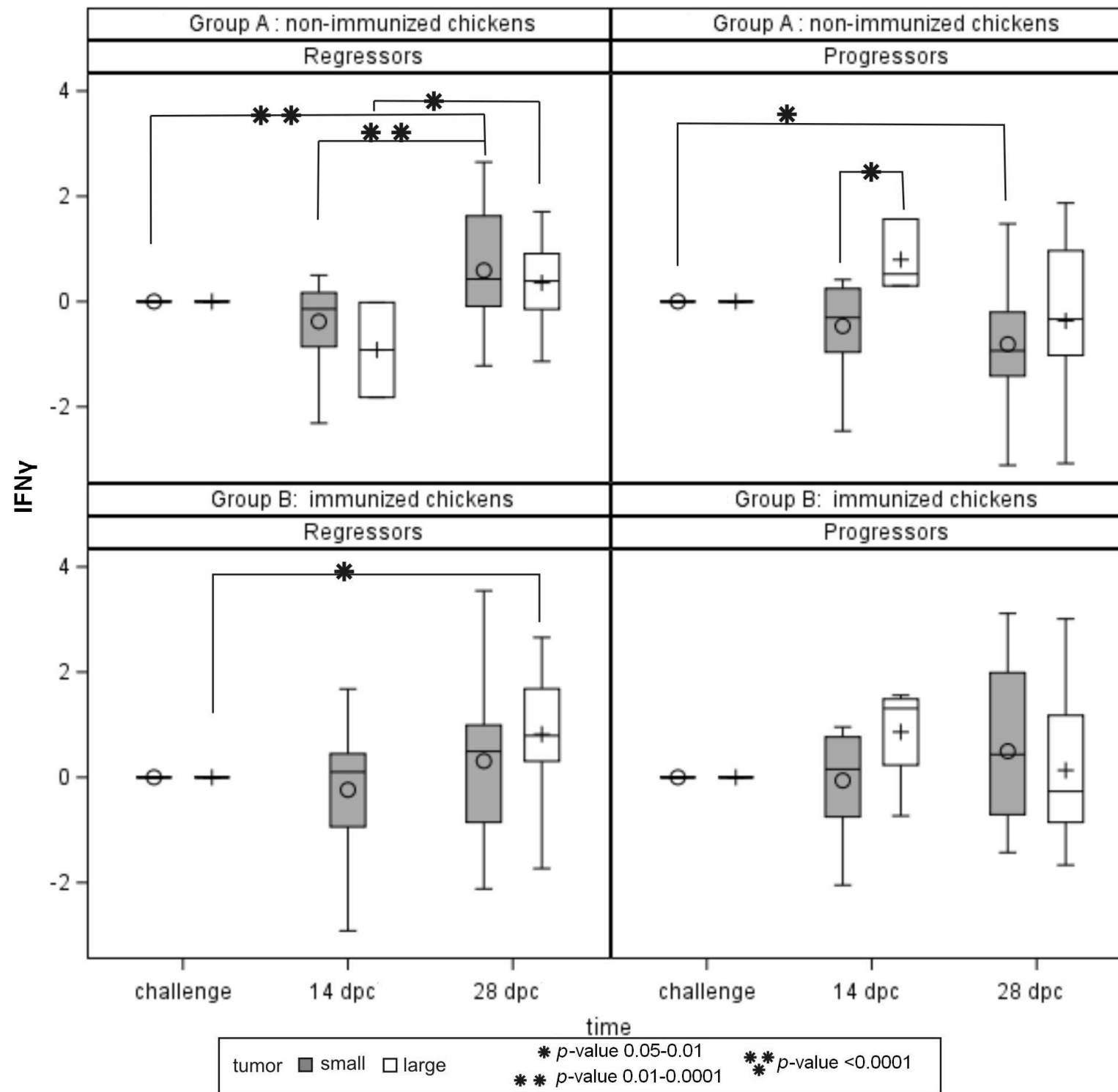

# IL 1

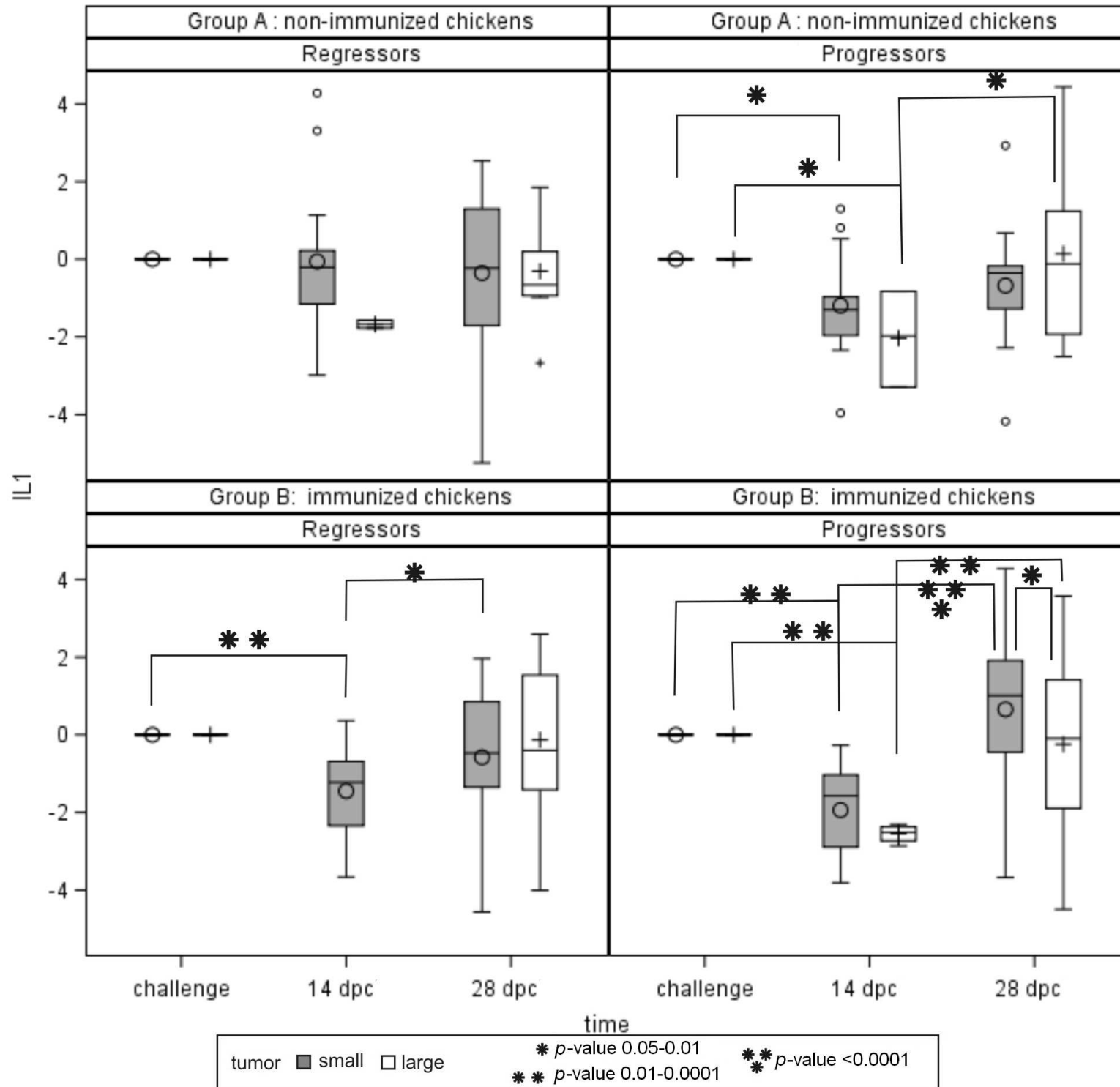

# IL2

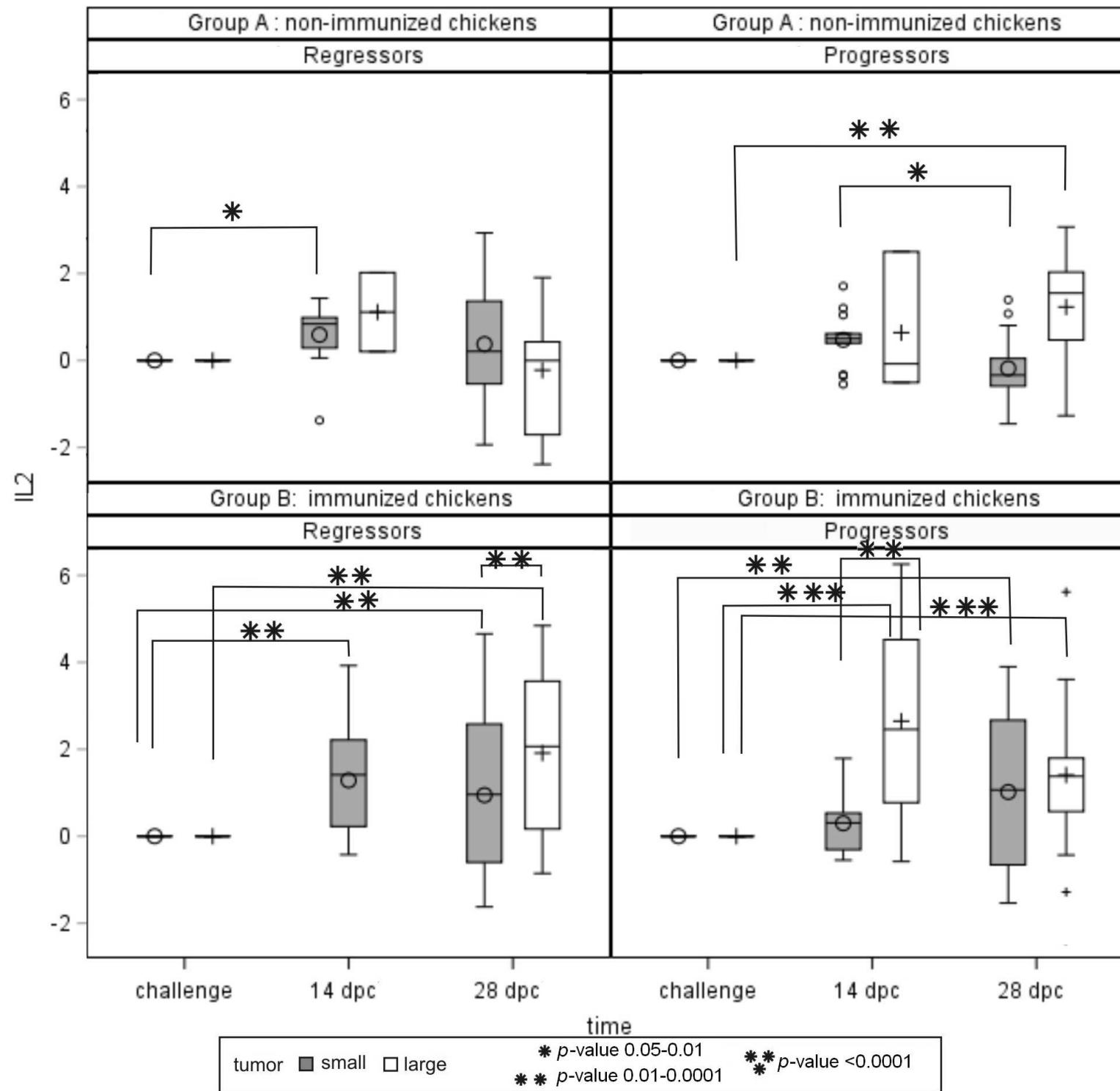

# IL4

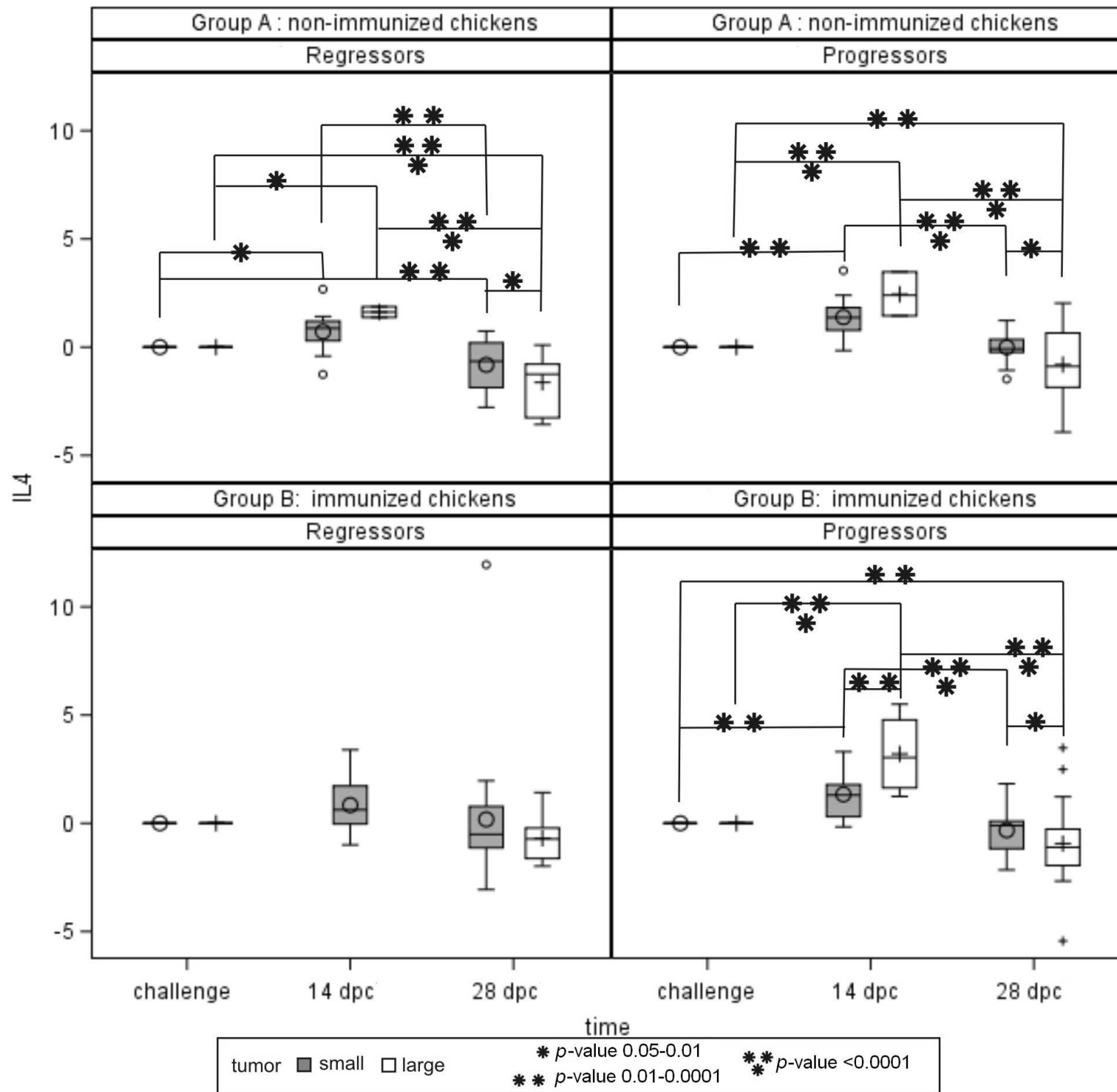

# IL8

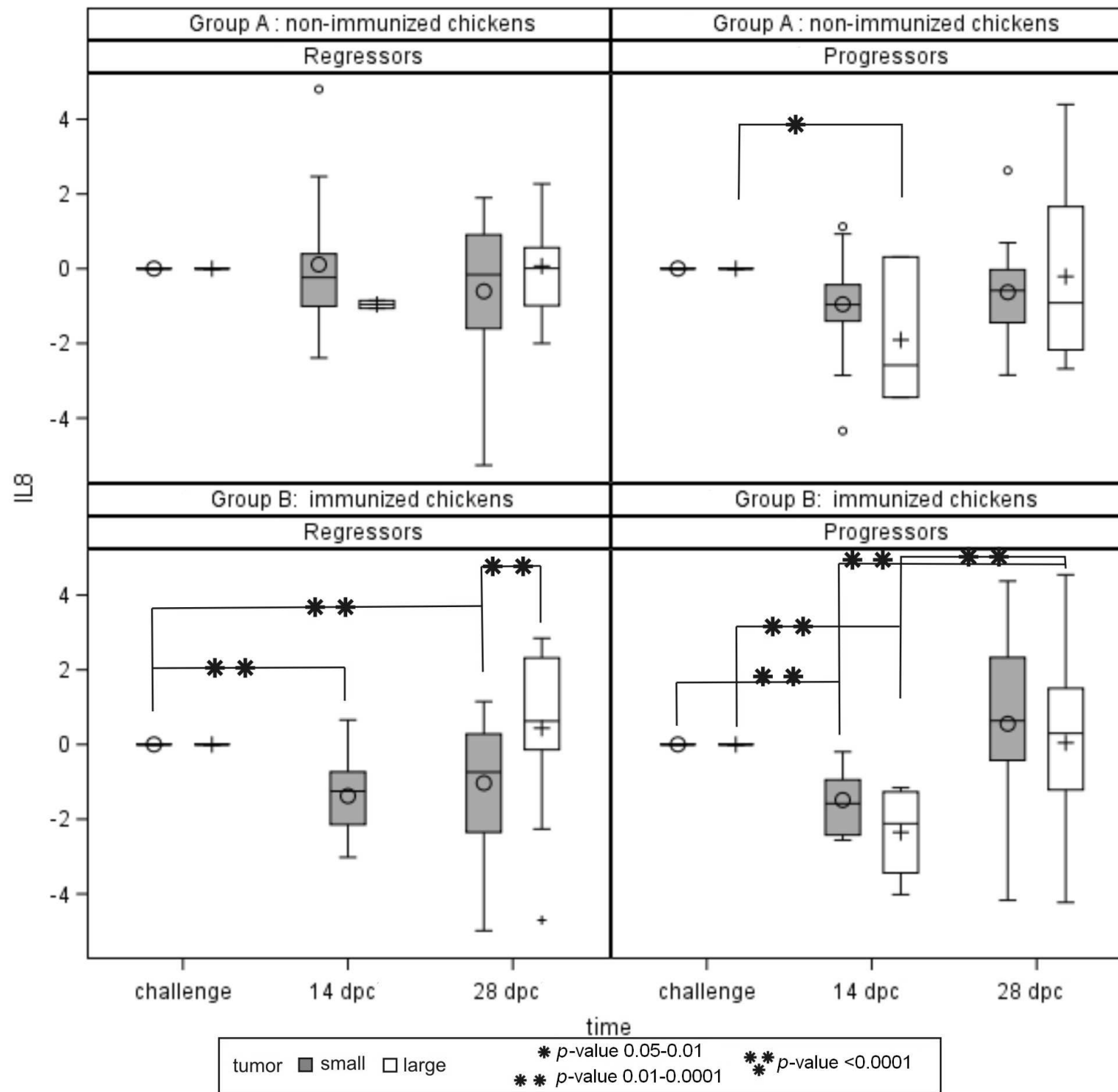

# IL10

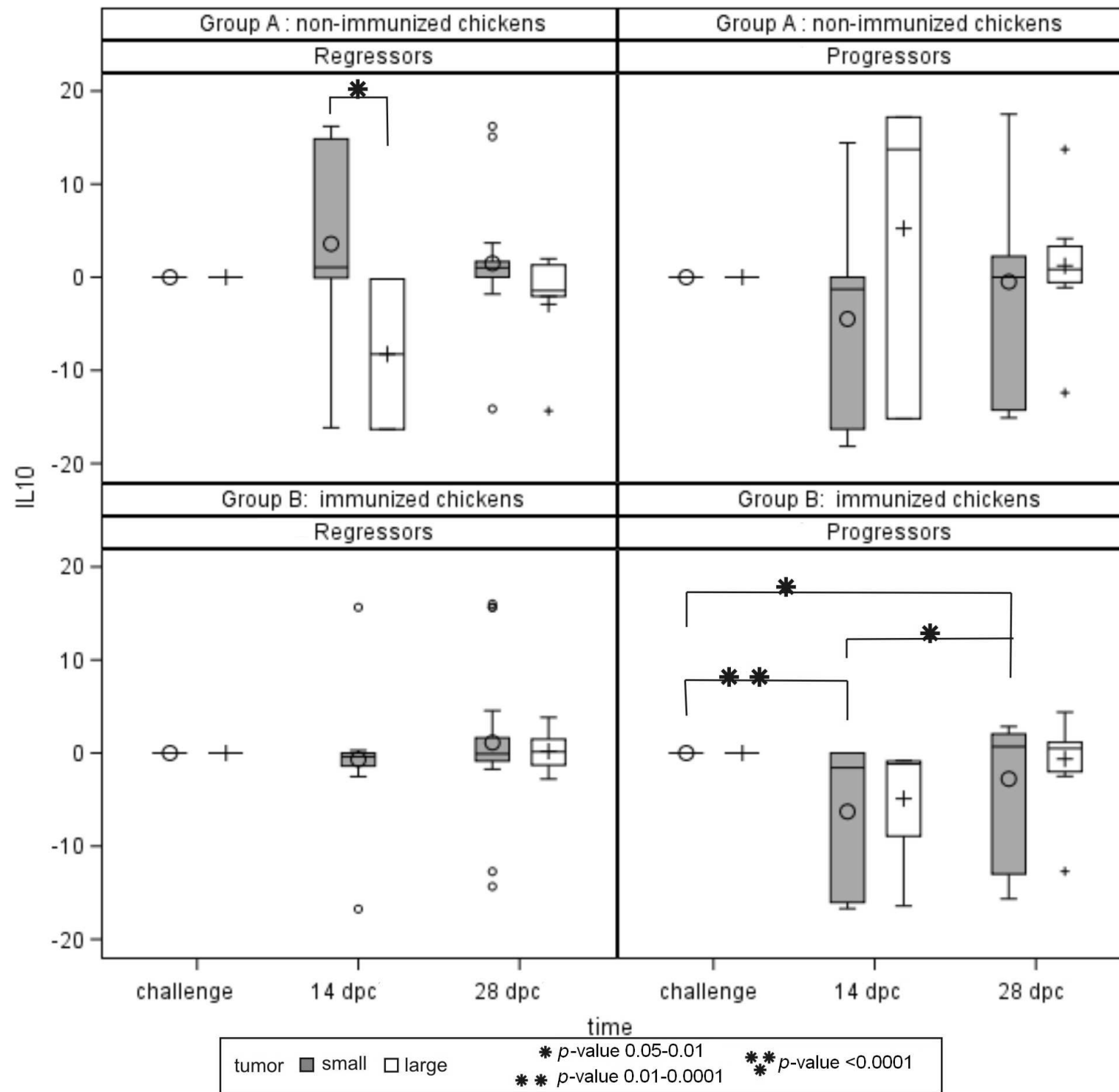

# IL12

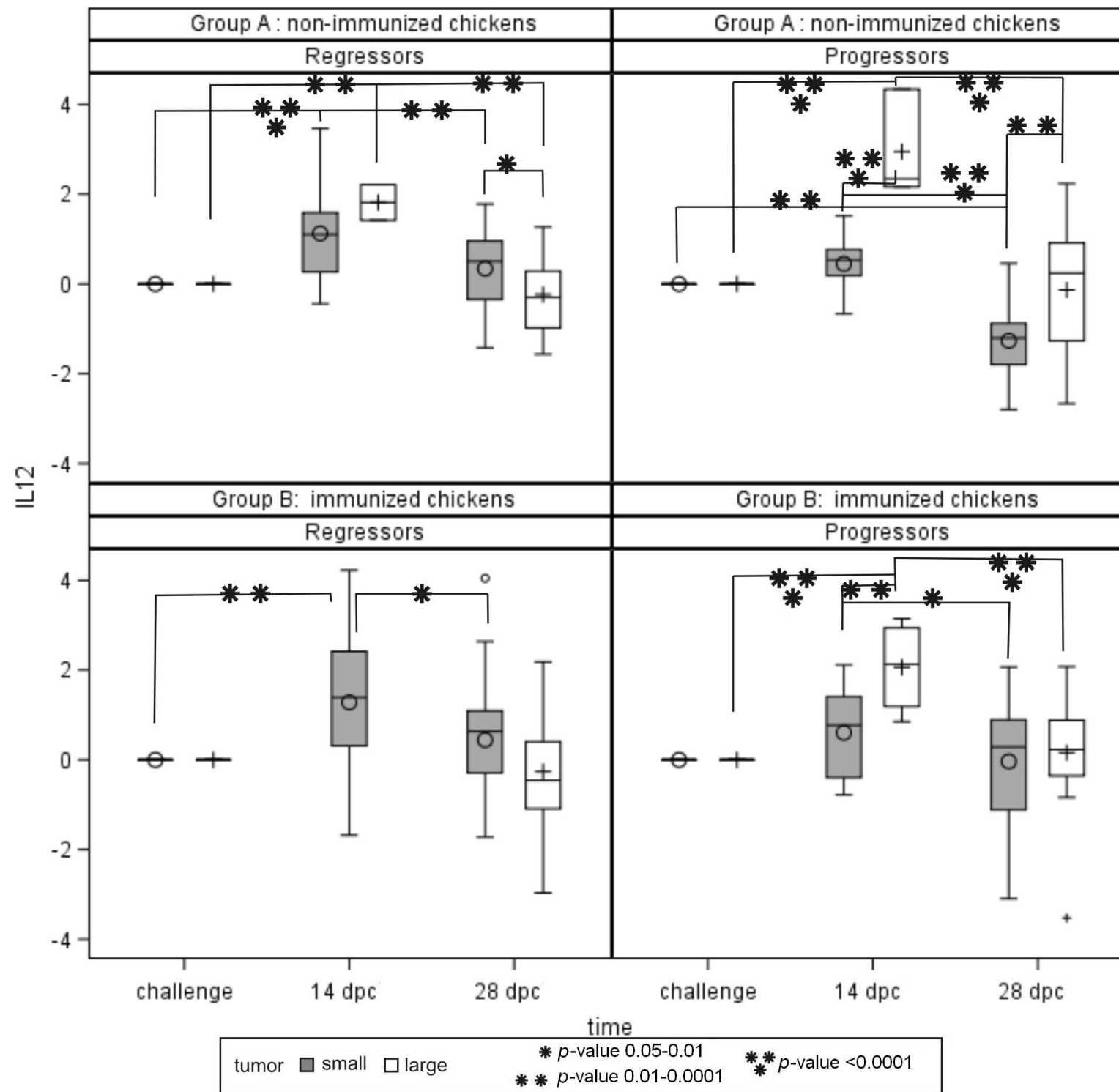

# IL15

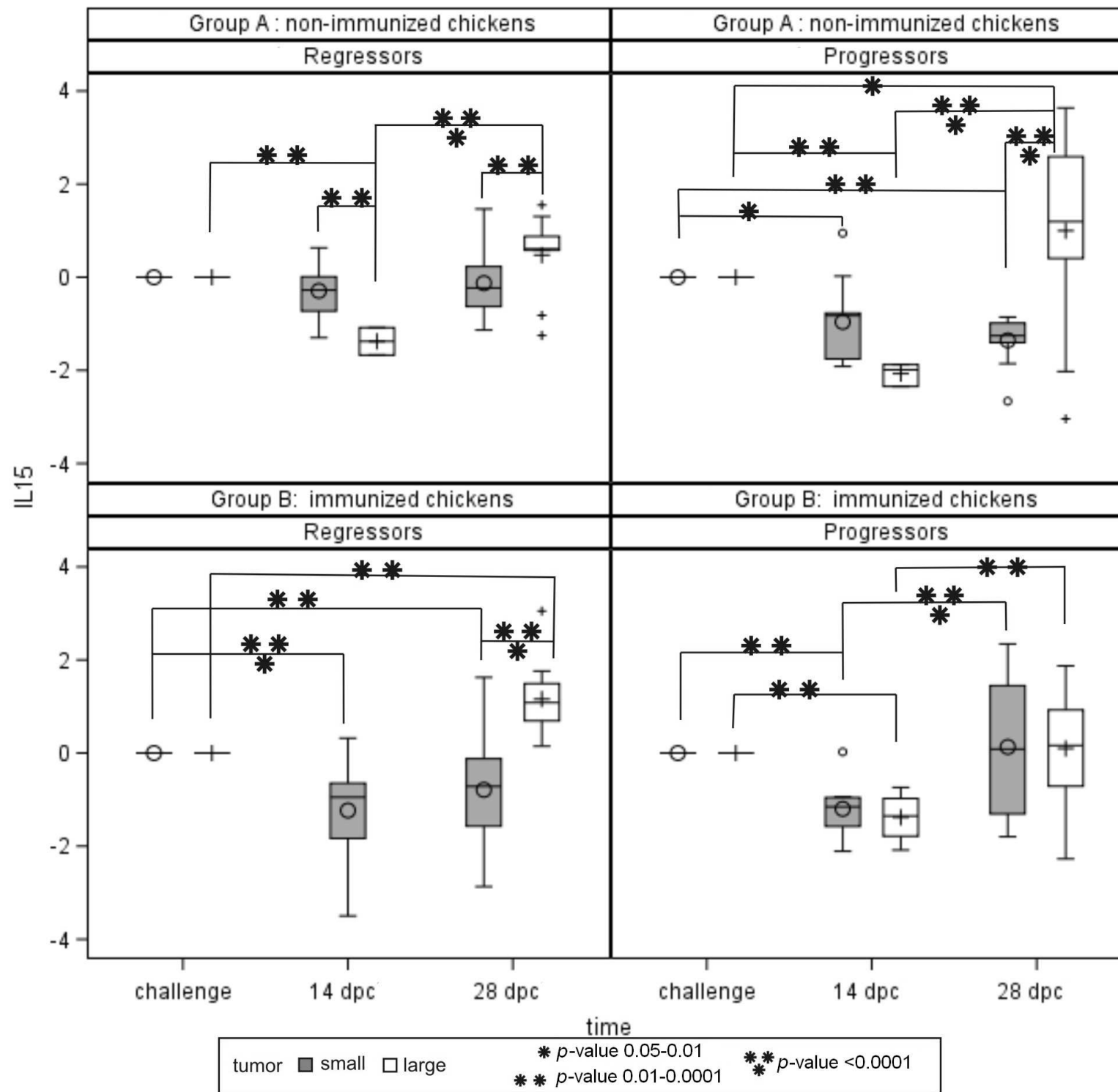

# IL17

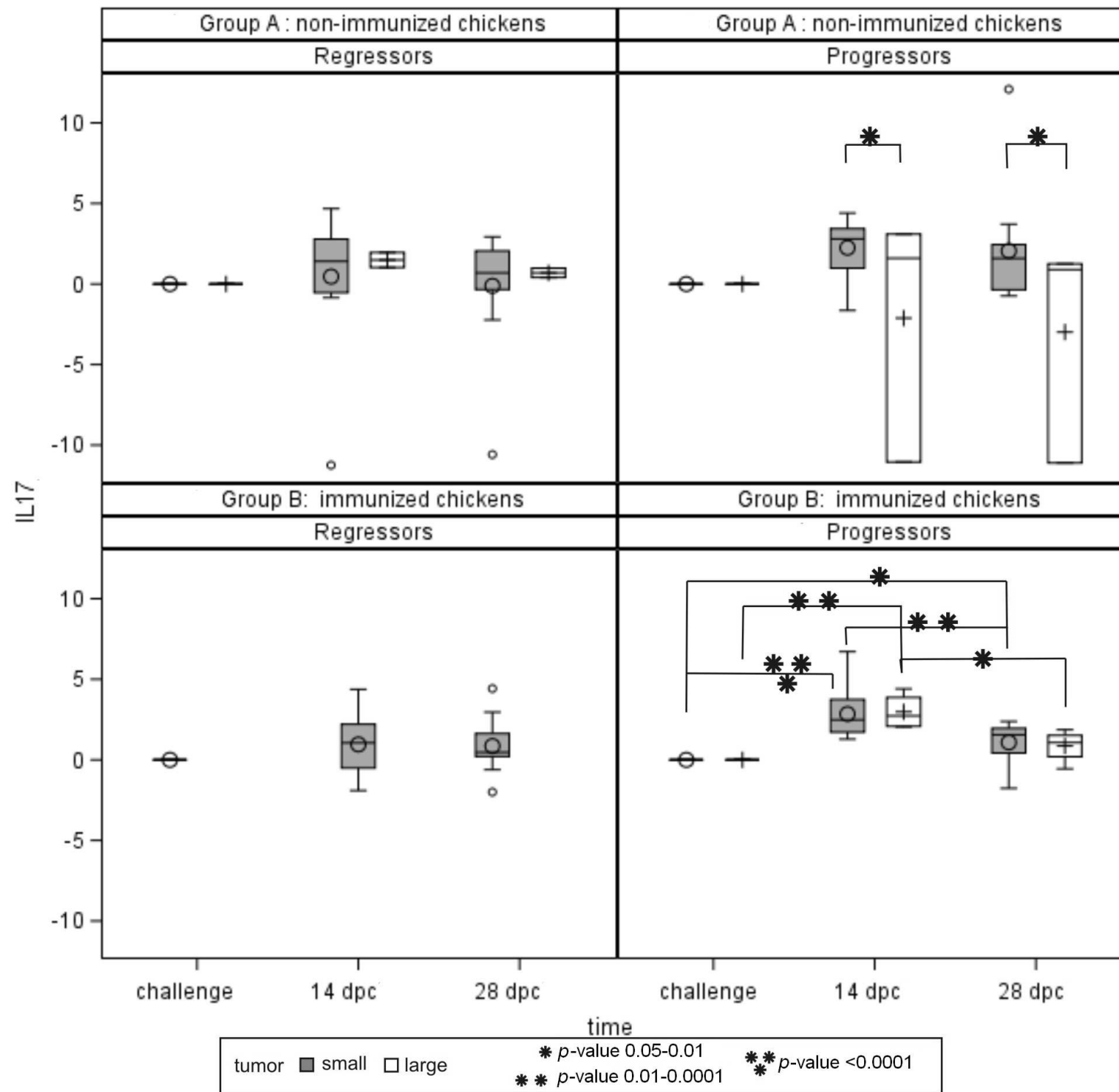

# IL18

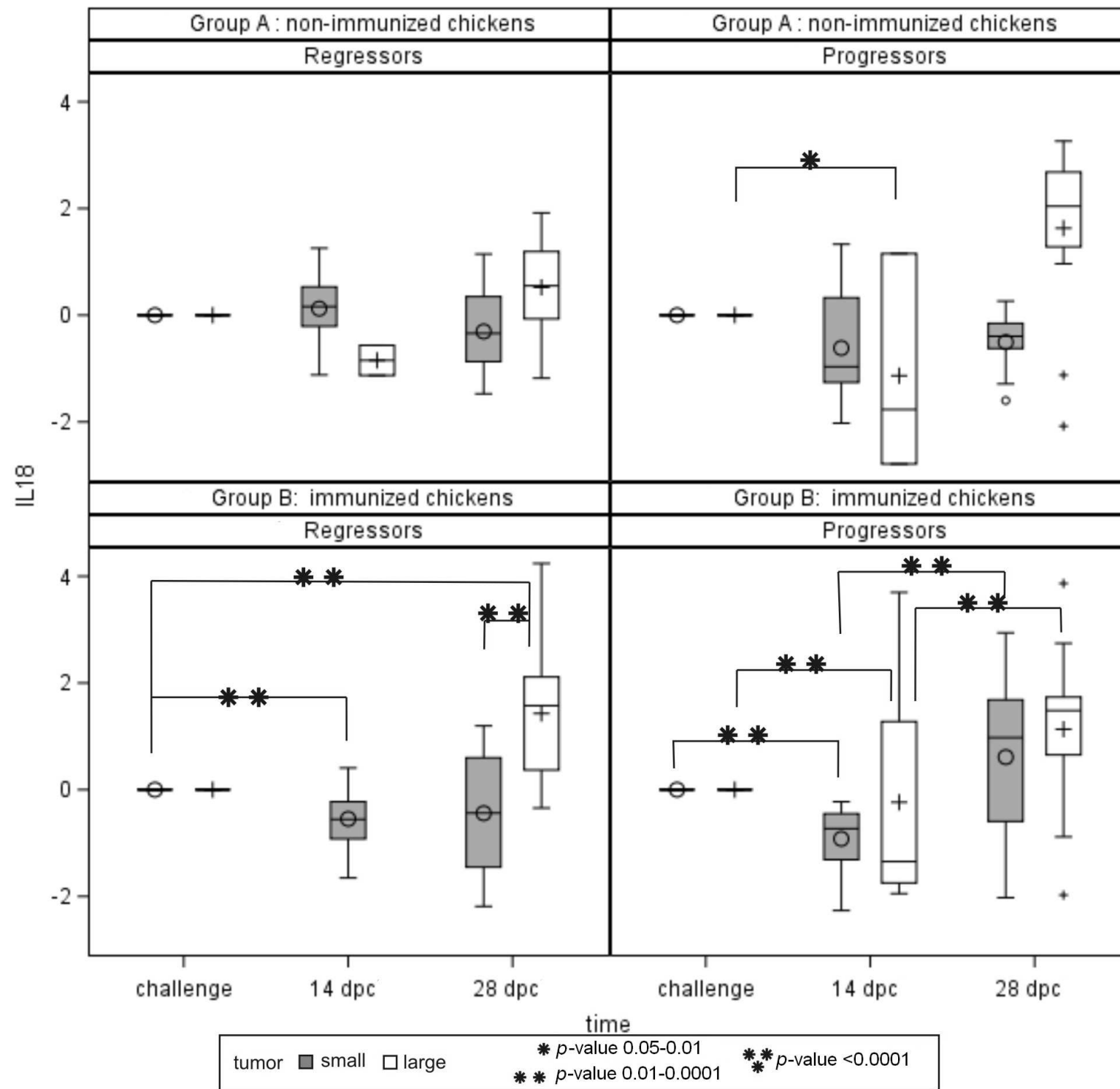

# iNOS

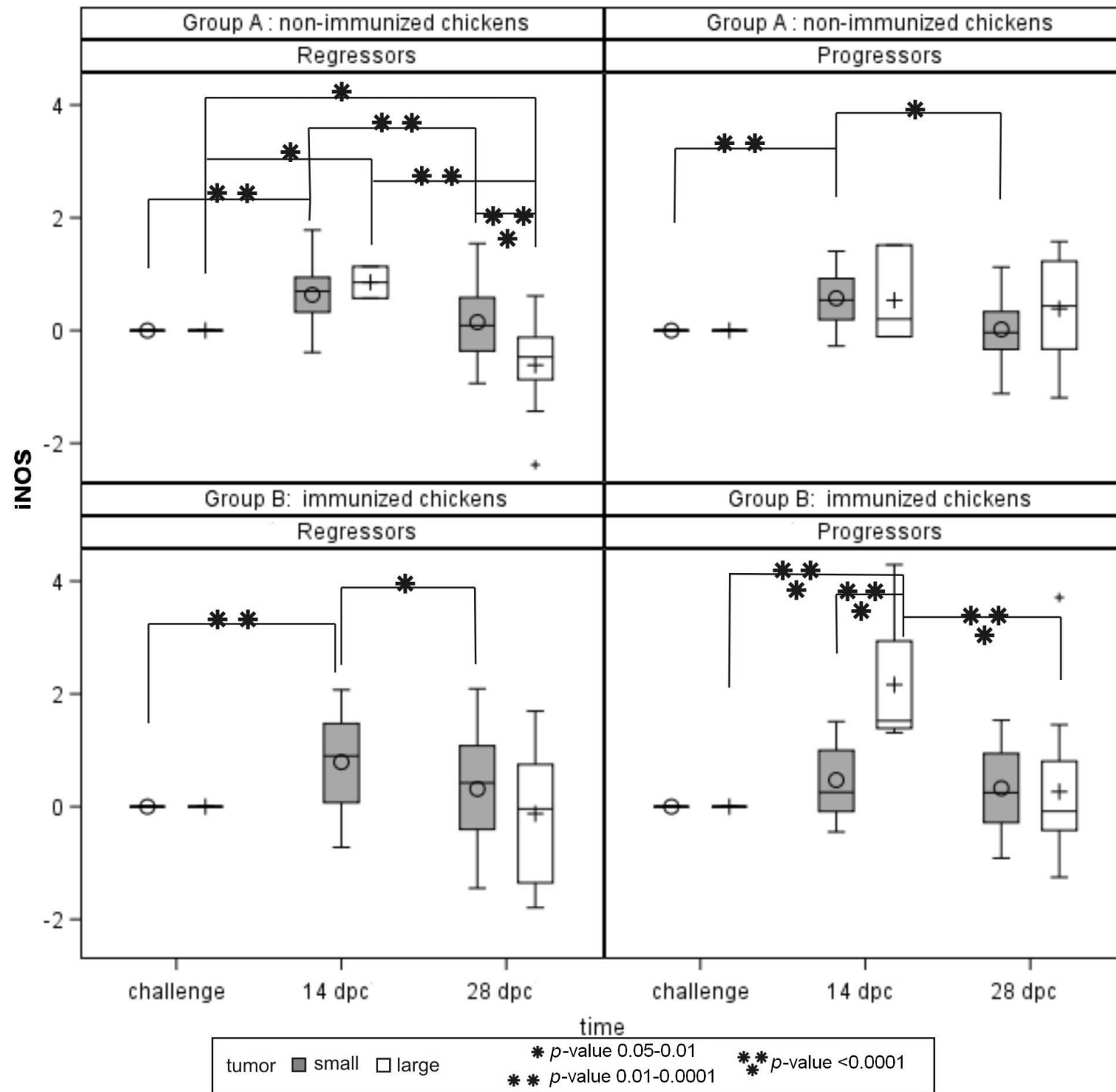

# LITAF

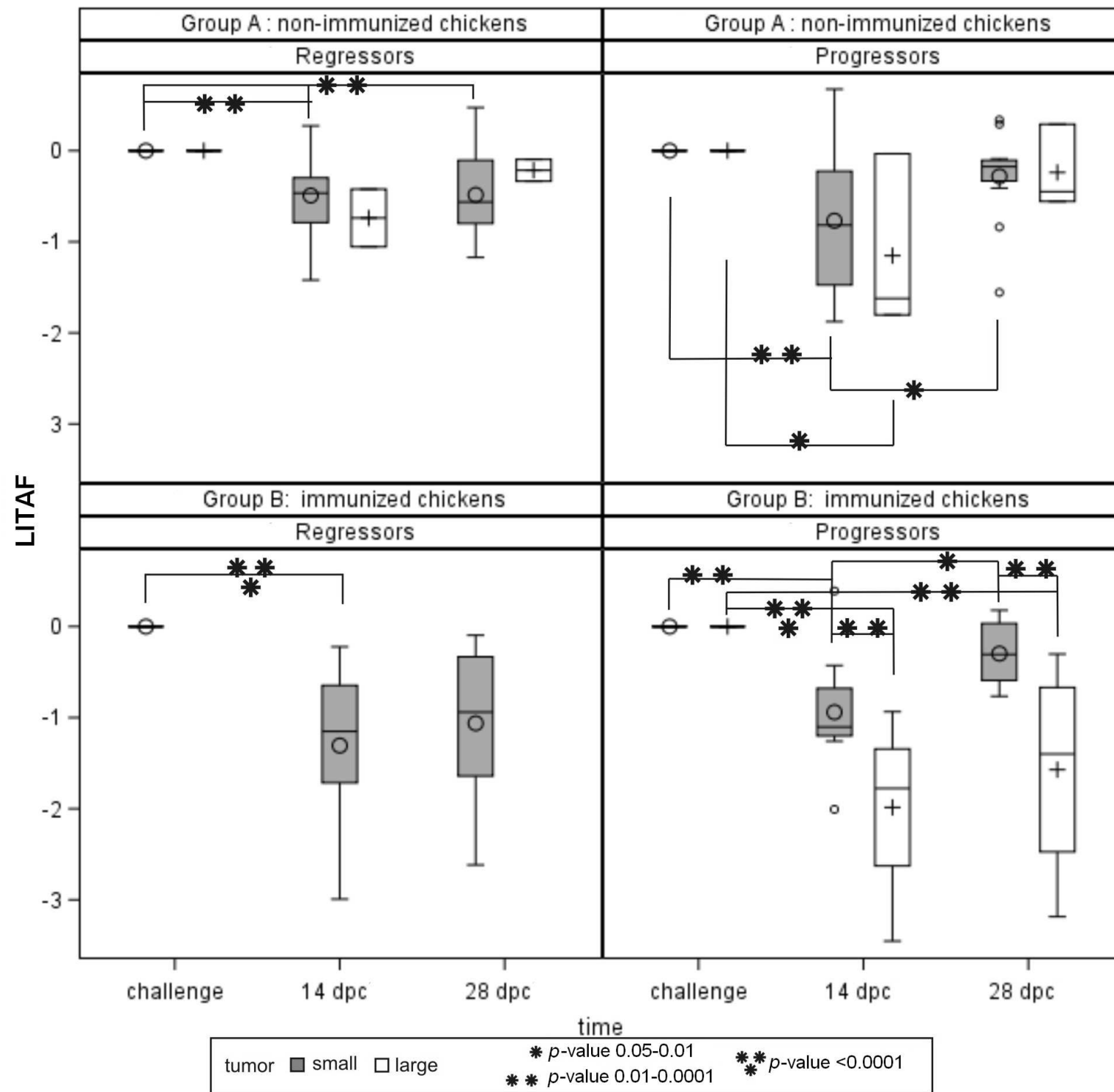

# MIF

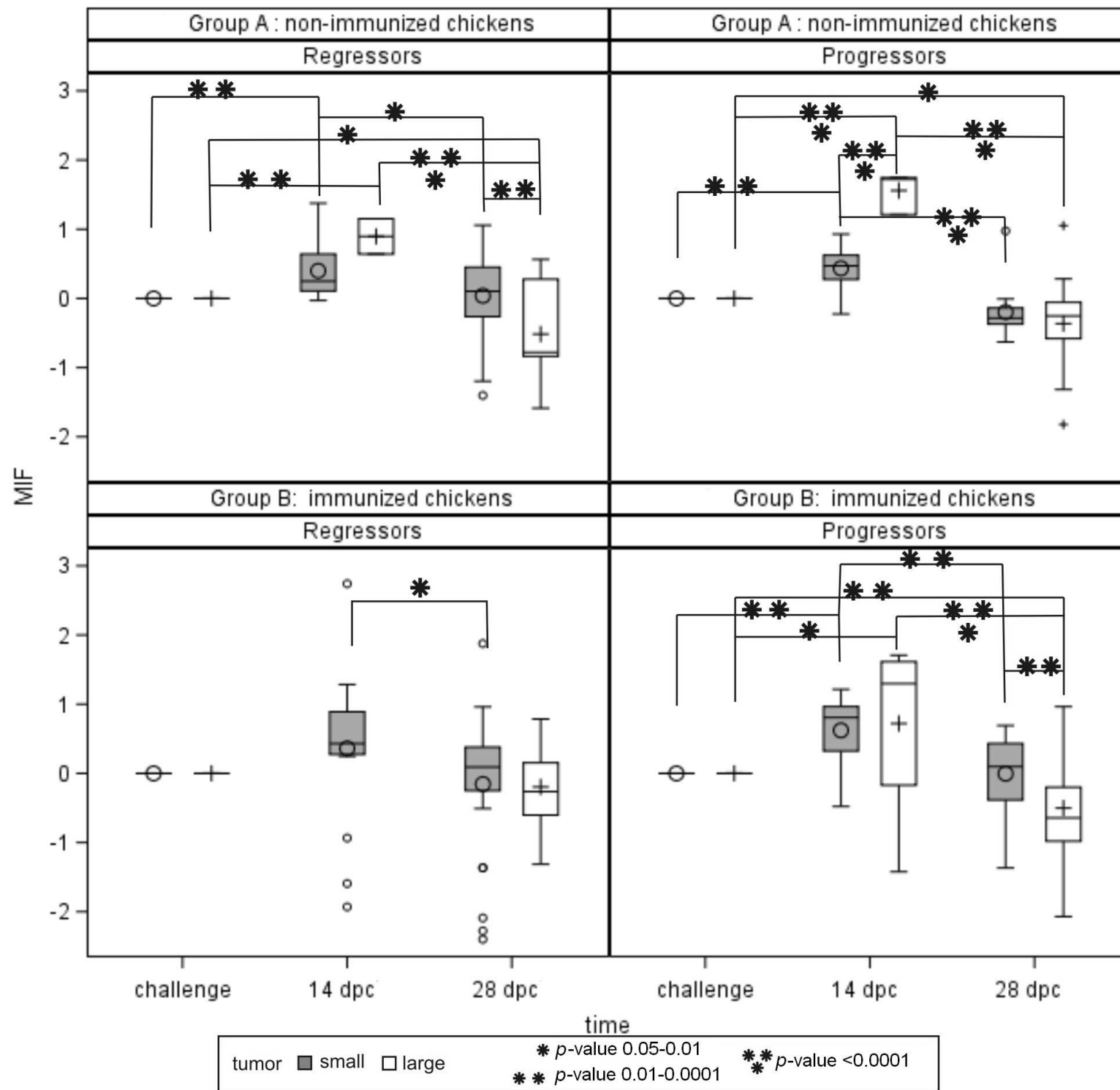

# TGFβ

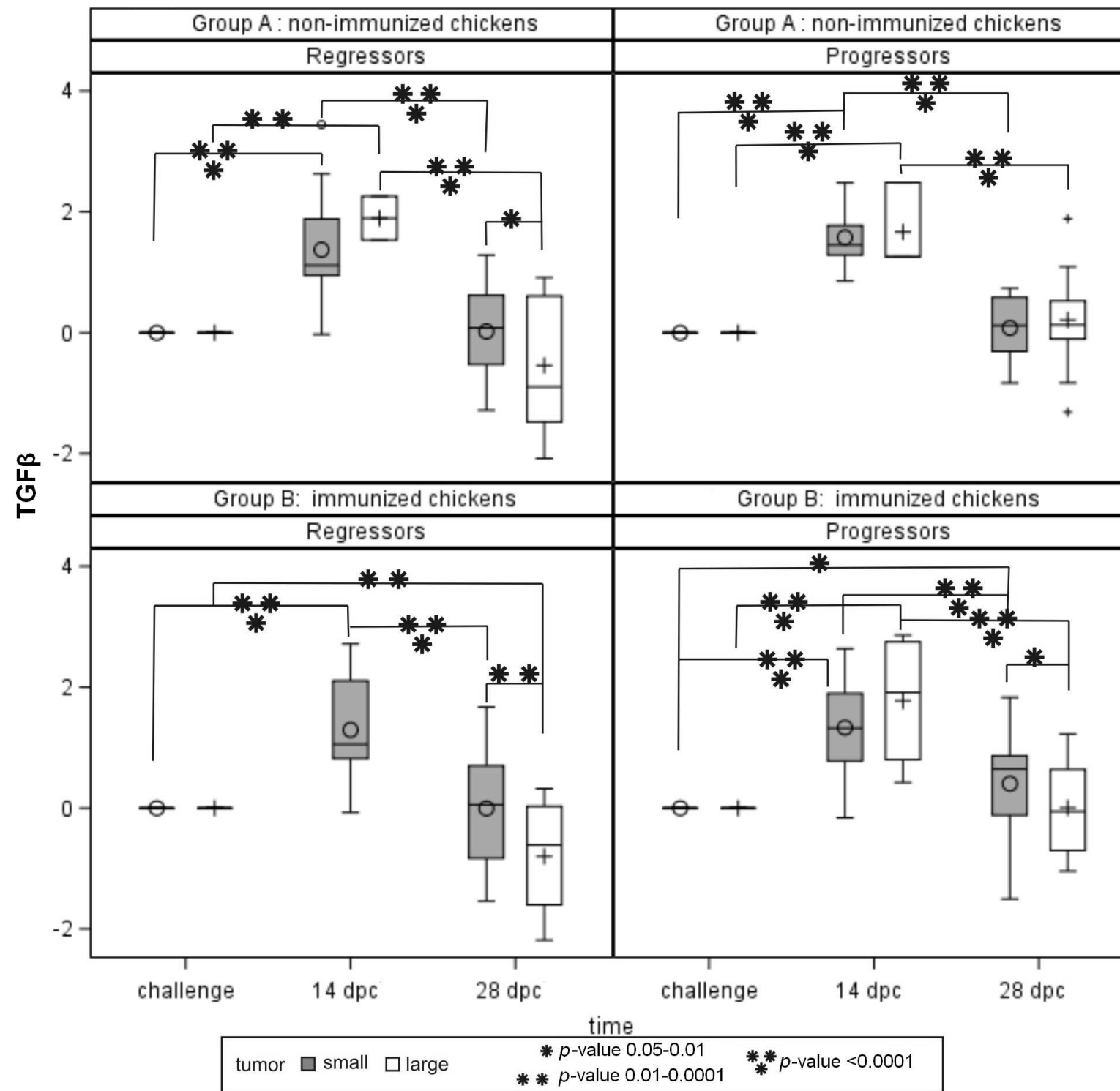

# TLR7

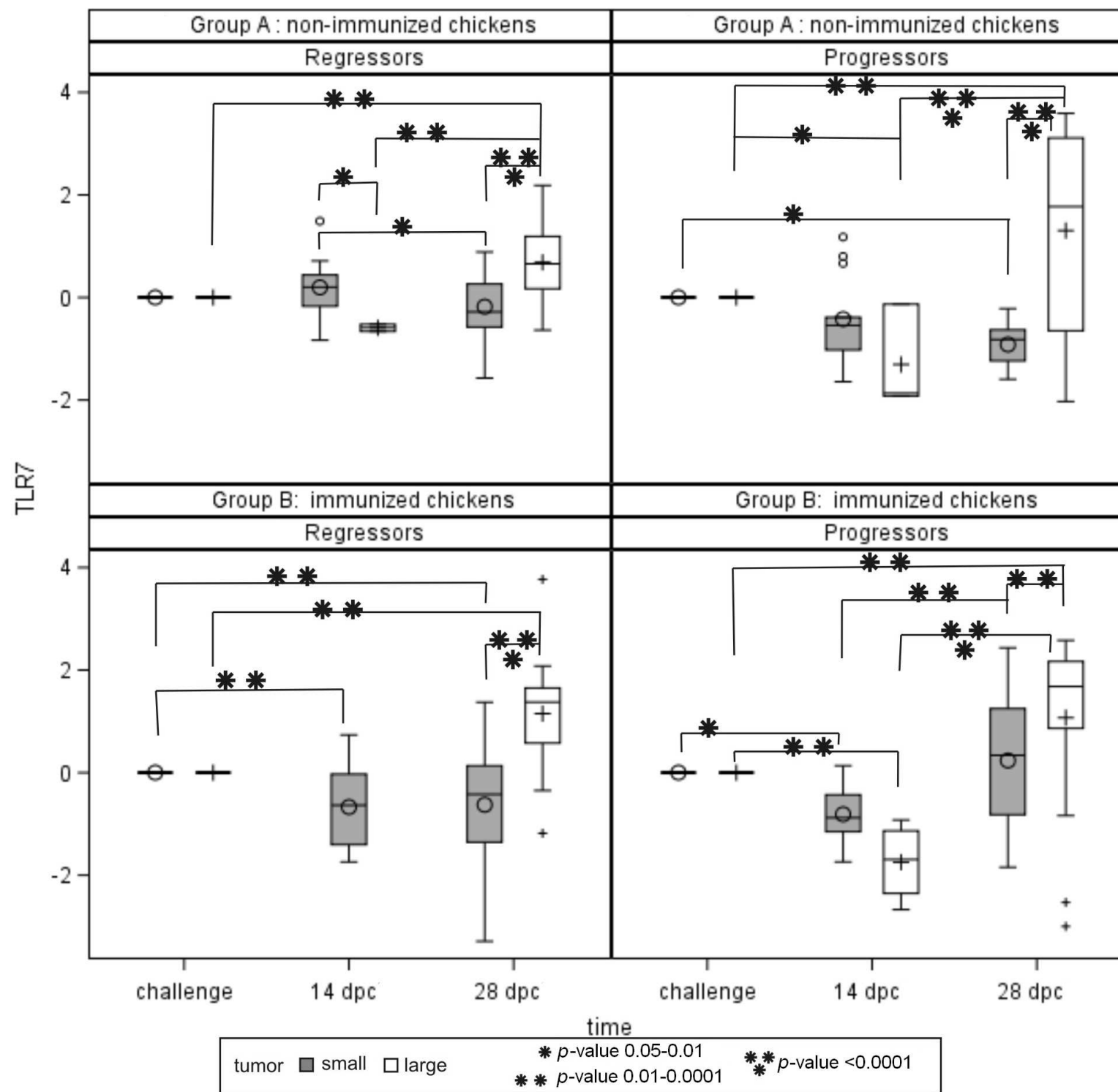

# TRAF5

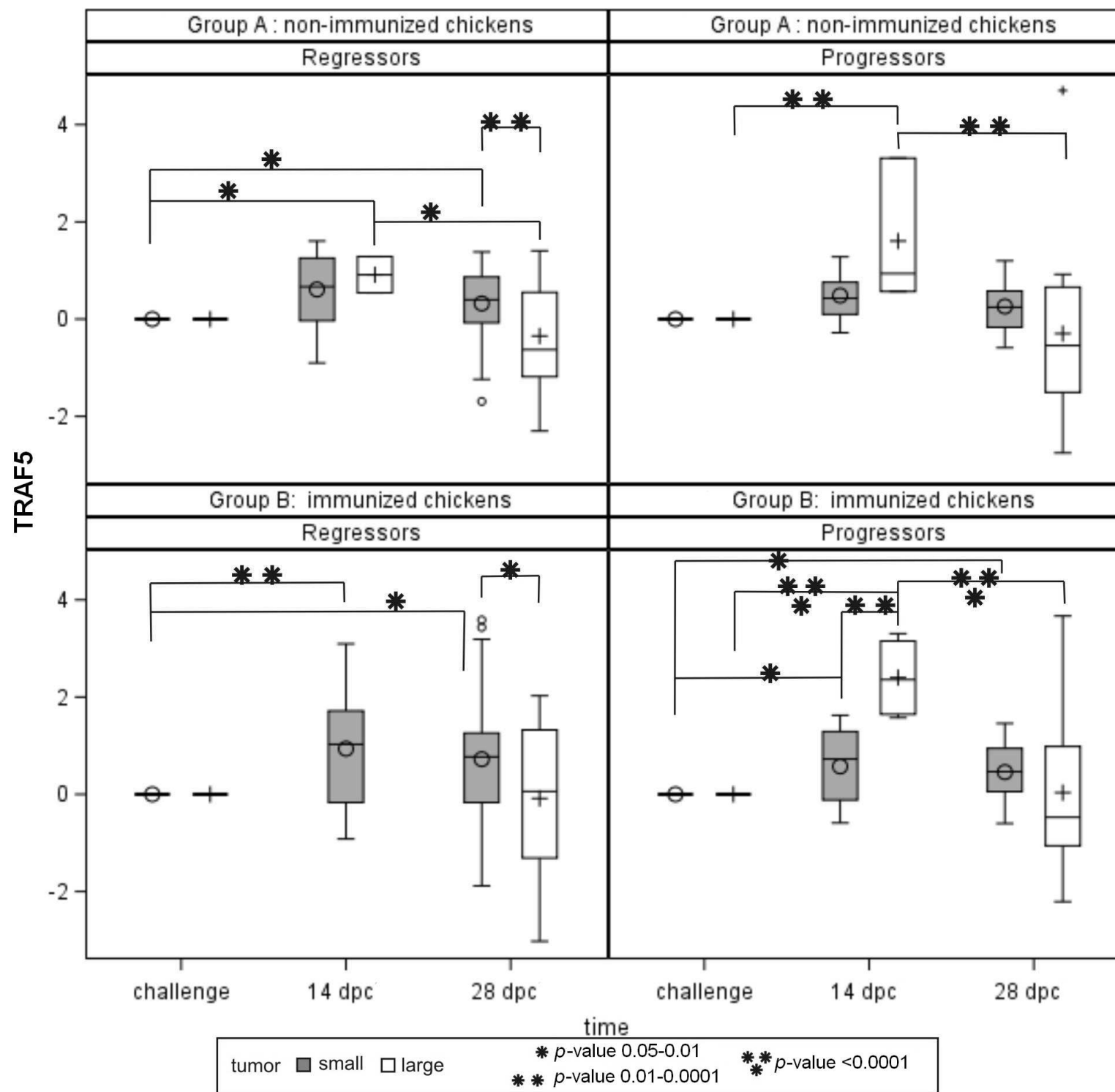

# XCL1

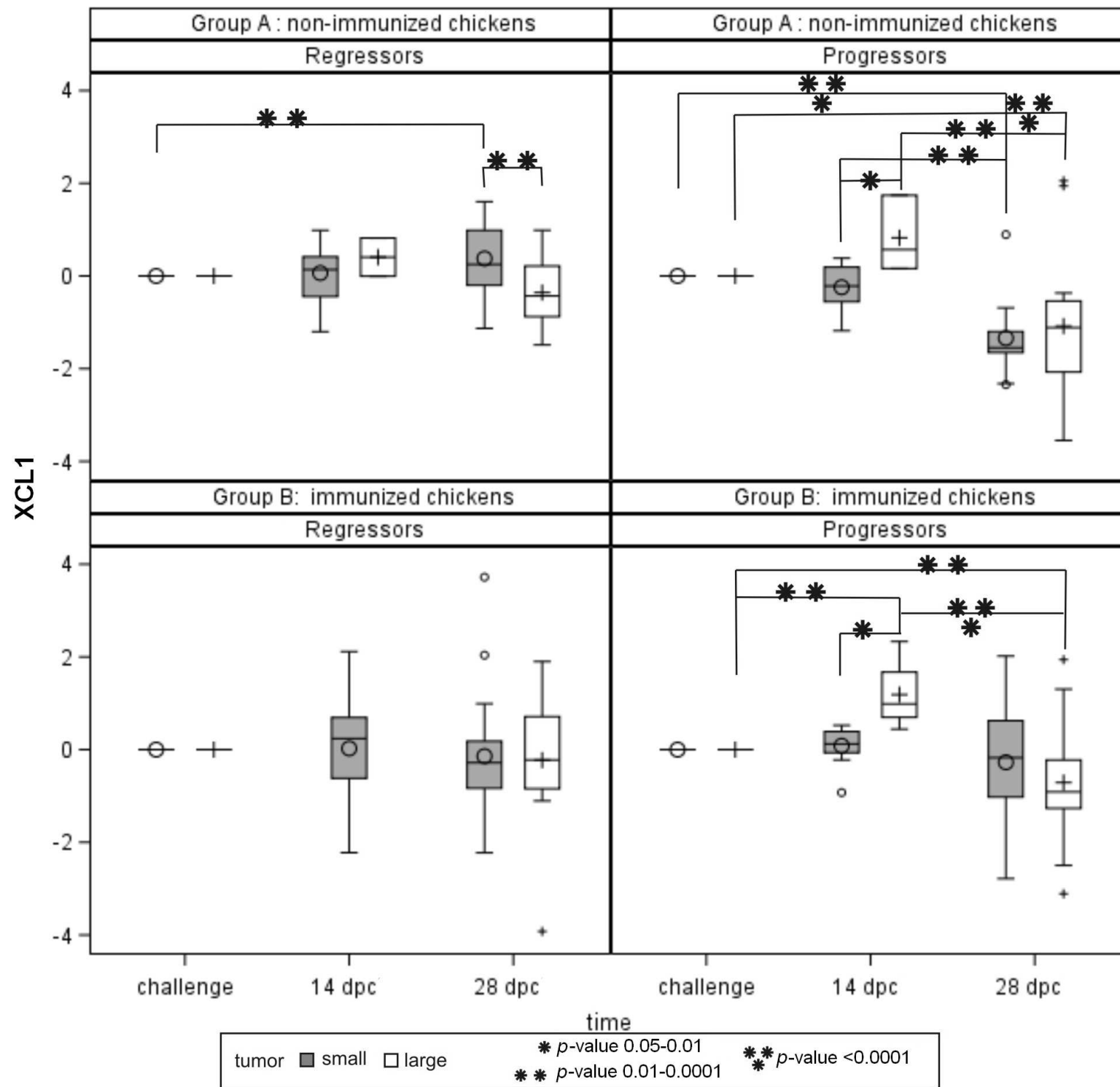

Supplement: Supplementary file 5 — Additional file 5. Cytokine profiles of selected genes in progressor and regressor groups of immunized and non-immunized chickens. These data show the expression profiles of all observed cytokines in progressor and regressor groups of immunized and non-immunized chickens. The expression value after challenge was normalized to the basic value of expression in the day of challenge. [file 13567_2017_423_MOESM5_ESM.pdf]
